# Mothers’ experiences of care pathways for children with speech and developmental concerns in Germany: A qualitative study of navigation, transitions, and communication

**DOI:** 10.1371/journal.pone.0357294

**Published:** 2026-09-16

**Authors:** Stephanie Rupp, Hanna Schwendemann

**Affiliations:** IU International University of Applied Sciences, Erfurt, Germany; Hacettepe University: Hacettepe Universitesi, TÜRKIYE

## Abstract

Mothers of children with speech, communication, or developmental concerns in Germany may experience complex pathways through a support system marked by multiple institutional interfaces. This qualitative interview study examined how mothers experience the care and support pathway from the first signs of concern to the uptake or coordination of support services, and which conditions they describe as burdensome or relieving. It constitutes the interview component of the mixed-methods project “EGUBE – Eltern gut begleitet?”. Eight semi-structured interviews from the ethically approved main study were included. Recruitment was open to parents and caregivers with parental responsibility irrespective of gender; however, the realised interview sample consisted exclusively of mothers. Interviews were conducted predominantly via videoconference, with one interview conducted by telephone; the children were school-aged or adolescents at the time of interview. Prior pilot interviews served only to refine the interview materials and were not included in the present analysis. Data were analysed using qualitative content analysis informed by Kuckartz and Rädiker, combining deductive and inductive elements. The broader health literacy perspective sensitised the analysis to questions of orientation, comprehensibility, navigation, coordination, communication, and organisational demands, while categories and subcategories were refined in close engagement with the interview material. A documented codebook and a case-by-category matrix supported transparent cross-case analysis. The findings reconstruct recurring nodal points along the pathway: first signs of concern, marked by mothers’ observations and/or feedback from daycare or kindergarten; diagnostic assessment as an ambivalent turning point, sometimes experienced as “too early” or only weakly clarifying, but at other times as providing orientation when a problem was named or classified; high demands related to navigation and organisation, including appointments, applications, and information-seeking; fragmented interfaces between institutions and professions; and transitions—especially into school—as coordination-intensive phases. Communication was described as central to orientation, ranging from supportive explanations to experiences of devaluing attributions. Resources such as social networks, insurance status, and language- or translation-related accessibility were identified as factors shaping both access and burden. Explicit markers of emotional distress appeared particularly in situations of limited responsiveness, attribution, or uncertainty, whereas relief was associated with experiences of fit, support, and restored capacity to act. These findings point to functional requirements for supportive structures, including reliable orientation and navigation support, clear and structured communication, and improved coordination across institutional interfaces, without presupposing the effectiveness of any specific model.

## Introduction

Child speech, communication, and other developmental concerns represent an important field of care in which early diagnostic clarification, evidence-based therapy, and interprofessionally coordinated support may be relevant for developmental, participation, and educational opportunities [1]. From parents’ perspectives, however, the pathway from first signs of concern to the uptake and coordination of support services is often far from linear. International studies on diagnostic and support services for children with developmental disabilities or developmental concerns describe these pathways as fragmented and shaped by barriers related to accessibility, continuity, information transfer, and intersectoral coordination [2,3]. Early intervention services, medicine, therapy, daycare settings, schools, and, where applicable, other services each involve their own responsibilities, communication logics, and access requirements.

In Germany, care pathways for children with speech, communication, and developmental concerns may involve several sectors and institutional entry points. Preventive child health examinations are usually conducted by paediatricians; the U9 examination, for example, takes place before school entry and may be relevant for identifying developmental concerns. Speech and language therapy and occupational therapy are typically accessed through medical prescription. For more complex developmental concerns, social paediatric centres (Sozialpädiatrische Zentren, SPZ) may provide interdisciplinary assessment and guidance. Early intervention services (Frühförderung) support children with developmental delays or disabilities and often operate at the interface of health, social, and educational services. Daycare centres, schools, and school-related support arrangements belong to the education sector and may become particularly relevant at transitions, especially around school entry. These institutional arrangements form the care context within which the pathways reconstructed in this study unfolded.

For families, this can create a care pathway that resembles less a linear sequence than a series of recurring situations of orientation, clarification, coordination, and transition. In this context, diagnostic and care trajectories for young children with developmental concerns have been described as involving repeated discontinuities between health and education contexts, insufficient continuity of information, and the experience of having to navigate a fragmented system without clear guidance [2]. At such interfaces, information loss, unclear responsibilities, and increased demands for coordination and navigation may become particularly relevant [2,3]. International and German-language studies further indicate that coordination demands, information demands, and unmet support needs may be highly relevant for families in complex child care contexts; this applies both to the coordination of required services and to dealing with health information under complex care conditions [4,5]. From a health services research perspective, the key question is therefore not only whether services are formally available, but also how care is organised in everyday practice, how it functions across interfaces, and whether it can actually be reached and used by those who need it [6]. Systematic reviews on family-centred care for children with special health care needs have further shown that partnership between families and professionals is associated in particular with better communication, greater satisfaction, improved access, and more efficient use of services [7]. A multiperspective study from Québec likewise identified barriers related to accessibility, continuity, and the validity of diagnostic services, and highlighted greater formalisation of procedures, improved information transfer, and forms of case navigation as key areas for improvement [2]. For the present context, the question is therefore not only whether support services exist, but also how accessible, comprehensible, and coordinable they become from families’ perspectives over the course of care [3].

What remains less well understood is how parents themselves interpret these pathways and work through them over time: when they first perceive a need for action, how they experience assessment and diagnostics, where orientation is gained or lost, how transitions are managed, and what role communication and available resources play. Studies on diagnostic trajectories of children with developmental disabilities or autism spectrum disorders show that parents often perceive the diagnostic phase as beginning with their first concerns about their child’s development—well before formal assessment—and that these early phases are frequently marked by barriers to access, problems of continuity, and uncertainty about next steps [3,8]. What is less clearly elaborated, however, is how such demands, from parents’ and caregivers’ perspectives, condense into a subjective process logic over the further course of care and support. A qualitative reconstruction from parents’ and caregivers’ perspectives can make an independent contribution here by rendering visible perceived demands for orientation, navigation, and coordination, conditions associated with burden and relief, and concrete support needs, without claiming to determine frequencies or objective quality of care.

For the theoretical framing of this study, health literacy is particularly useful when understood not in exclusively individualistic terms, but in terms of the interaction between individual abilities and the demands of care contexts. In a widely cited definitional core, health literacy refers to the ability to access, understand, appraise, and apply health-related information [9]. Beyond this, health literacy has been conceptualised as a multidimensional resource for action that extends beyond merely functional information processing and also includes interactive and critical capabilities [10]. It has also been emphasised accordingly [11] that health literacy cannot be reduced to individual reading ability, but must be considered in relation to the demands of a complex care system. Relational perspectives develop this idea further by conceptualising health literacy as the outcome of an interactive and context-dependent interplay between individual preconditions, communication, and the care context [12].

This perspective is especially applicable to complex child care constellations. Parents frequently act on behalf of their child, must bring together information from different institutional contexts, and make decisions under conditions in which responsibilities, procedures, and next steps are not always self-evident. Accordingly, the central question shifts away from whether parents possess sufficient individual competencies and toward what demands care systems and organisations place on families, and to what extent these demands are designed in ways that are understandable, manageable, and supportive of action.

This leads to the concept of organisational health literacy. It emphasises that organisations and care systems should design information, communication, and procedures in ways that enable people to orient themselves and to find, understand, and use relevant information and services [13,14]. A scoping review of criteria for health-literate health care organisations highlights communication with service users, as well as easy access and navigation, as key organisational fields of action [15]. Practical implementation strategies for such health literacy-sensitive design have been described, among others, in universal precautions approaches, which structure information, communication, and care processes in ways that make them understandable, accessible, and manageable for patients and caregivers without assuming that comprehension difficulties will necessarily be obvious in individual cases [16]. For the present research question, this opens up a framework for reading parental accounts of navigation, comprehensibility, responsibilities, and interface coordination not as expressions of individual inadequacy, but as indicators of demands and possible mismatches within the care system.

Linked to this perspective is the assumption that parents in complex care constellations take on substantial tasks of navigation, coordination, and decision-making. Depending on the course of events, the available resources, and the surrounding system conditions, this role may be experienced as relieving or burdensome. There is evidence that care coordination and unmet support needs are relevant for families in child care contexts [5]. Work on health literacy likewise suggests that burdens should not be understood solely as individual coping problems, but also analysed in relation to information demands, the design of information, organisational conditions, and the complex contextual conditions of care [4]. In addition, differences in resources, for example with regard to prior knowledge, social networks, or linguistic accessibility, may shape how effortful, manageable, or relieving care is experienced. For families with limited language proficiency, the availability of professional interpretation may be an important condition for understandable and safe care, although the literature has described this primarily in other paediatric care contexts [17].

In this context, communication is not merely an accompanying feature of care, but a process dimension through which orientation, interpretation, cooperation, and capacity for action are actively produced. Health literacy-sensitive information and counselling accordingly emphasise the importance of understandable, audience-appropriate communication and active checks of understanding, for example through teach-back and chunk-and-check approaches [16,18]. Qualitative studies involving parents of children with rare diseases further underline that communication with health professionals is experienced as particularly robust when transparency, communicative openness, and trustworthiness are evident [19]. Therapeutic contexts likewise suggest that patients’ evaluations of care are substantially shaped by communication and relationship quality, comprehensive information, explanations, and attention to individual needs [20]. The focus of the present study, however, is not the objective measurement of communicative quality, but the reconstruction of mothers’ experiences of how communication at relevant interfaces is described as supportive, orienting, irritating, or burdensome.

Against this background, the present paper reports the qualitative interview component of the mixed-methods project “EGUBE – Eltern gut begleitet?”. The aim of this study is to reconstruct mothers’ perspectives on their child’s care and support pathway, from the first signs of concern to the uptake or coordination of support services. Recruitment for the interview component was open to parents and caregivers with parental responsibility irrespective of gender; however, the realised interview sample consisted exclusively of mothers. The focus therefore lies on the non-linear process logic of care pathways as reconstructed by mothers, processes of orientation and clarification, navigation and coordination demands over time, communicative experiences at interfaces, and conditions that mothers describe as relieving or burdensome. The paper thus does not primarily investigate presumed parental deficits, but rather the fit between families’ information, navigation, and coordination demands on the one hand, and the communicative and organisational conditions of the care system on the other. The quantitative online survey and the mixed-methods integration will be reported separately.

This qualitative interview study addresses the following questions:

Question 1: How do mothers experience their child’s care and support pathway in the context of speech, communication, and developmental concerns, and which conditions of the care system do they describe as relieving or burdensome?

Question 2: Which navigation, coordination, and decision-making tasks do mothers take on over the course of care, and in which situations do they report associated burden or relief?

Question 3: How do mothers describe the role of communication and collaboration at interfaces between daycare, medicine, therapy, education, and authorities in relation to orientation, access, and continuity of care?

Question 4: Which support needs and wishes do mothers identify, and which functional requirements for supportive services can be derived from these accounts?

## Methods

### Study design and reporting

This qualitative interview study was embedded within the mixed-methods project “EGUBE – Eltern gut begleitet?” and aimed to reconstruct experiences of the care and support pathway from the first perception of child-related concerns to the uptake or coordination of support and therapy services. The interview component was open to parents and caregivers with parental responsibility for children with parent-reported speech, communication, and/or developmental concerns. The realised interview sample consisted of eight participants, all of whom were mothers. Reporting was guided by the Consolidated Criteria for Reporting Qualitative Research (COREQ) [26], and the completed checklist is provided as S5 File. Quotations are identified by interview ID and, where documented in the transcript, by time stamps; where time stamps were not available for individual passages, source locations are indicated by a content-based positional anchor.

### Recruitment, sample, and data collection

Following ethics approval, recruitment was conducted through professional networks, professional associations, personal contacts with institutions in the fields of occupational therapy and speech and language therapy, and social media channels. The sample was assembled in a pragmatic yet purposive manner. Eligible participants were parents or caregivers with parental responsibility for children with parent-reported speech, communication, and/or developmental concerns who were receiving occupational therapy and/or speech and language therapy at the time of the survey or had received such services within the previous two years. Recruitment was therefore directed at parents and caregivers with parental responsibility irrespective of gender. In the realised interview sample, however, all eight participants were mothers; this was taken into account when interpreting and wording the findings. Recruitment for the main study began with the anonymous online survey on 30 April 2024; interviews included in the present analysis were conducted between July and October 2024. Twenty-two survey respondents provided their email address and thereby indicated their willingness to be contacted for an interview. These contact details constituted an opt-in pool for possible follow-up interviews rather than a complete sampling frame from which all eligible families could be systematically followed up. Interviews were organised sequentially within the pragmatic scope of the qualitative interview component. The realised sample covered variation in child age and parent-reported developmental concerns, while remaining limited to mothers who had entered or recently used occupational therapy and/or speech and language therapy services. Eight interviews were completed as part of the ethically approved main study.

Prior to ethics approval for the main study, two voluntary preliminary pilot interviews were conducted to test and refine the interview aid and practical procedures. The pilot participants were informed about data protection and their rights and provided written and oral consent, including permission to use the pilot conversations for instrument development and scientific purposes. They were informed that participation was voluntary and could be discontinued at any time without giving reasons and without any disadvantages. During the pilot phase, the pilot interviews were reviewed and preliminarily coded in order to test the interview and analytic procedures. However, they were not included in the final analytic dataset of the present study; no pilot quotations, case material, or pilot-derived findings are reported in this manuscript. Because recruitment was open and distributed across multiple channels, a conventional response rate based on all individuals reached cannot be validly determined. Reasons for non-participation or dropout among the remaining contacts were not systematically collected. The characteristics of the interview sample are summarised in Table 1.

**Table 1:** Aggregate characteristics of the qualitative interview sample (n = 8).

Characteristic	Aggregate description
Interview participants	Mothers, n = 8
Child age group at the time of interview	School-aged children and adolescents
Parent-reported diagnosis or developmental concern	Speech- or language-related diagnosis or concern, n = 6; broader neurodevelopmental diagnosis or concern, n = 3

*Note. To minimise re-identification risk in this small qualitative sample, age information is reported only in broad aggregate form, and no participant-level characteristic profiles are presented in this table. Diagnostic and developmental-concern categories were not mutually exclusive. Diagnoses and concerns were reported by participants and were not clinically verified.*

The realised sample comprised eight mothers; the children were school-aged or adolescents at the time of interview. Diagnoses or developmental concerns were documented as contextual information based on mothers’ reports and were not clinically verified. Although the study protocol primarily targeted parents of preschool-aged children, the open age entry in the online survey resulted in a realised sample comprising mothers of school-aged children and adolescents. This variation was retained and reported transparently because the analytical focus was not on age-specific comparisons, but on reconstructing the reported care and support pathway. Retrospective accounts of earlier phases were therefore treated as part of the subjective reconstruction of the process.

Seven of the eight interviews included in the present analysis were conducted via videoconference; one interview was conducted by telephone at the participant’s request. Interviews lasted between 19 and 68 minutes, with a median duration of 39 minutes. No repeat interviews were conducted, and transcripts or findings were not returned to participants for comment.

The interview guide was piloted prior to the ethically approved main study. In the pilot version, an emotion diagram was used as a conversational aid. Based on the pilot experience, this element was replaced in the main study by a chronology table sent to participants in advance in order to better support the reconstruction of key stages along the care pathway. The table was intended solely as an optional private preparation aid; completed copies were not collected or analysed and did not form part of the study data. The content of the guiding questions remained unchanged in its substantive orientation. Data from the pilot phase were not included in the present analysis. The interview guide, chronology table, and accompanying study materials are documented in S3 File.

Audio and video recordings were transcribed and de-identified. Transcription was initially carried out using f4x, and transcript quality was subsequently checked by listening to the recordings and corrected where necessary.

Interviews were conducted and transcribed in German. Analysis was carried out on the German transcripts. Quotations selected for the manuscript and Supporting Information were translated into English by one author after analysis and checked against the German original by the second author. Translation focused on preserving the analytic meaning of the excerpts while ensuring readability in English and protecting participant confidentiality. Where literal translation would have produced misleading or overly identifying wording, the translation was adjusted to convey the meaning of the original passage as closely as possible.

### Data preparation and analysis

Data analysis followed qualitative content analysis informed by Kuckartz and Rädiker [25]. The analytic approach combined deductive and inductive elements. The research questions and the broader health literacy perspective sensitised the analysis to issues of orientation, comprehensibility, navigation, coordination, communication, and organisational demands. At the same time, the category system was not applied as a fixed deductive health literacy coding framework. Instead, categories and subcategories were iteratively developed, refined, and differentiated in close engagement with the interview material.

The analysis proceeded in several interrelated steps. First, the transcripts were imported into MAXQDA; the software was used to manage the category system, document coding decisions, and support an exemplary coding comparison. Initial coding was guided by the research questions, the interview guide, and sensitising concepts derived from the health literacy and care pathway literature. During case-by-case analysis, the coding guide was iteratively refined, and empirically grounded subcategories were added or adjusted where the material required further differentiation. Based on the final coding guide, the interviews were analysed case by case, and the resulting coding profiles were brought together in a case-by-category matrix in order to make recurring patterns, variations, and deviant cases systematically visible in cross-case comparison.

Over the course of the analysis, the category system and key findings were repeatedly discussed in interdisciplinary research contexts and in professional exchange formats. These exchanges served to critically sharpen the categories, enhance the plausibility of interpretations, and reflect disciplinary perspectives, but they did not replace the case-based analysis, team discussions, or consensual coding work. We do not claim saturation in a strong quantitative or exhaustive sense. Rather, within the pragmatic scope of the qualitative interview component, the completed interviews provided information-rich material for the focused process-oriented analysis, and no further interviews were conducted once the central process-related categories recurred across cases and the coding guide no longer required substantial modification during consensual coding. A short version of the category system is presented in Table 2; the full version, including subcategories, definitions, and anchor examples, is documented in S1 File.

**Table 2:** Main categories of the codebook (short definitions).

Main category	Short definition
Professionals/institutions	Professionals, services, and institutions mentioned along the child’s care pathway.
Emotional well-being	Mothers’ subjective emotional responses to the care situation.
Interdisciplinary collaboration	Experiences with coordination and alignment between different professional groups and institutions.
Resources	Family-related, social, personal, or everyday resources experienced as helpful or burdensome.
Organisational barriers/critique	Structural challenges, lack of clarity, and points of criticism within the support and care system.
Communication	Experiences of communication with professionals and institutions.
Contextual conditions	Structural conditions that facilitate or hinder care and access.
Wishes	Explicitly stated wishes for improving support, information, and guidance.
Clinical picture	Descriptions of the problem situation, observed difficulties, and the diagnostic process.

To ensure transparency in the cross-case comparison, the coded main categories for each interview were documented together with supporting excerpts in a case-by-category matrix (S2 File). In addition, case-related contextual information, such as key institutions or structural conditions, were recorded for descriptive contextualisation but were not treated as independent results categories. The presentation of the findings does not follow a schematic step-by-step treatment of individual main categories. Instead, in the cross-case comparison, several thematically related categories and subcategories were condensed into four overarching analytical units in order to present recurring stages, decision situations, and process logics of the reported care and support pathway in a manner that was both appropriate to the subject matter and reader-oriented. The mapping of this analytical condensation is shown in Table 3.

**Table 3:** Analytical condensation of categories in the presentation of results.

Overarching analytical unit	Predominantly included categories/subcategories
Early perceptions and triggers of the care pathway	Clinical picture (especially early noticed difficulties, diagnostic process), professionals/institutions (e.g., daycare centre, social paediatric centre, paediatrician), communication
Assessment and access as an ambivalent phase of orientation	Clinical picture (especially unclear condition, diagnostic process, complex problem situation), organisational barriers/critique (especially lack of clarity in the support system, waiting times/bureaucracy), communication, emotional well-being
Navigation, coordination, and communication along the pathway	Interdisciplinary collaboration (successful/negative), communication (positive/negative), organisational barriers/critique (especially parental role responsibility, system responsibility, changing professionals), professionals/institutions, contextual conditions, emotional well-being
Transitions, resources, and future perspectives	Resources (positive/negative, social support), contextual conditions (facilitating/hindering), wishes (especially a fixed contact person, information needs), emotional well-being (especially future concerns), organisational barriers/critique, professionals/institutions (especially school)

The table shows how multiple main categories and subcategories were condensed, in cross-case comparison, into the four overarching analytical units used in the presentation of the findings. This mapping serves to enhance transparency regarding the logic of the results presentation; individual categories may therefore contribute to more than one analytical unit.

### Quality assurance and reflexivity

The interviews were conducted by the two principal investigators themselves. Both researchers were therapeutically trained and experienced in professional interviewing. This professional background was treated as a resource and as a potential source of interpretive bias. It supported sensitivity to therapeutic pathways, communication at care interfaces, and system-related demands, but it may also have shaped the interview interaction and interpretation, for example by encouraging participants to use professional terminology, to assume shared background knowledge, or to foreground system- and therapy-related experiences. These assumptions were reflected throughout the research process and discussed both within the team and in exchanges with external colleagues.

Several strategies were used to strengthen reflexivity and analytic traceability. During interviewing, open prompts were used to invite participants’ own process reconstructions, and the interviewers explicitly asked about both burdensome and supportive experiences in order to avoid a one-sided focus on deficits or burden. During analysis, emerging interpretations were repeatedly discussed, checked against the interview material, and documented through a coding guide, case-by-category matrix, and supporting excerpts. The pilot phase informed the refinement of the interview aid and sensitised the researchers to process-related themes relevant to the subsequent main study, but pilot data were not included in the present analysis.

Coding was carried out by the two researchers on the basis of the coding guide and followed a consensus-based interpretive logic. Discrepancies in coding, category boundaries, or segmentation were discussed in several rounds of consultation, and the category system was refined where necessary. In addition, an exemplary coding comparison was conducted in which 50% of one interview was coded by both coders and percentage agreement was calculated after minor segmentation differences had been excluded as disagreements. The resulting agreement of approximately 90% was used as a supplementary consistency check within the project. It is not reported as a measure of strictly independent reliability, because it was based on a limited subset of the material and embedded in an iterative, consensus-based analytic process; accordingly, no chance-corrected reliability coefficient was calculated. The combination of documented coding decisions, consensual discussion, case-by-category comparison, linked supporting excerpts, and supplementary materials served to enhance the intersubjective traceability of the analytic process.

### Ethics and data protection

Ethical approval for the main study was granted by the Ethics Committee (Ethikkommission) of IU Internationale Hochschule on 22 April 2024; no ethics committee reference number was assigned, as this is not standard procedure of the institutional Ethics Committee. The overall EGUBE study was registered in the German Clinical Trials Register (DRKS00034313). The interview study reported here was conducted between July and October 2024, after ethics approval. Participation in the main study was based on informed written consent, including consent to audio/video recording and the use of de-identified quotations in publications. The study was conducted in accordance with relevant ethical principles for research involving human participants. Authors had access to participants’ contact details for recruitment and scheduling purposes only; transcripts and analytic materials used for this manuscript were de-identified, and the authors did not retain direct identifiers in the analysis dataset. The de-identified analytic dataset underlying this manuscript corresponds to the minimal dataset described in the Data Availability Statement and provided as Supporting Information. Direct identifiers were removed, and potentially identifying contextual details were generalised or omitted prior to publication. Full interview transcripts are not publicly shared because the detailed narrative accounts may still allow indirect identification through combinations of contextual information. Data protection officers were involved in the study planning. Data were stored in a secure research environment in accordance with the General Data Protection Regulation (GDPR); access rights and deletion periods were defined.

Publicly available study materials include the interview guide, codebook, case-by-category matrix, de-identified evidence tables, and the completed COREQ checklist provided as Supporting Information (S1–S5 Files).

## Results

**Fig 1:**
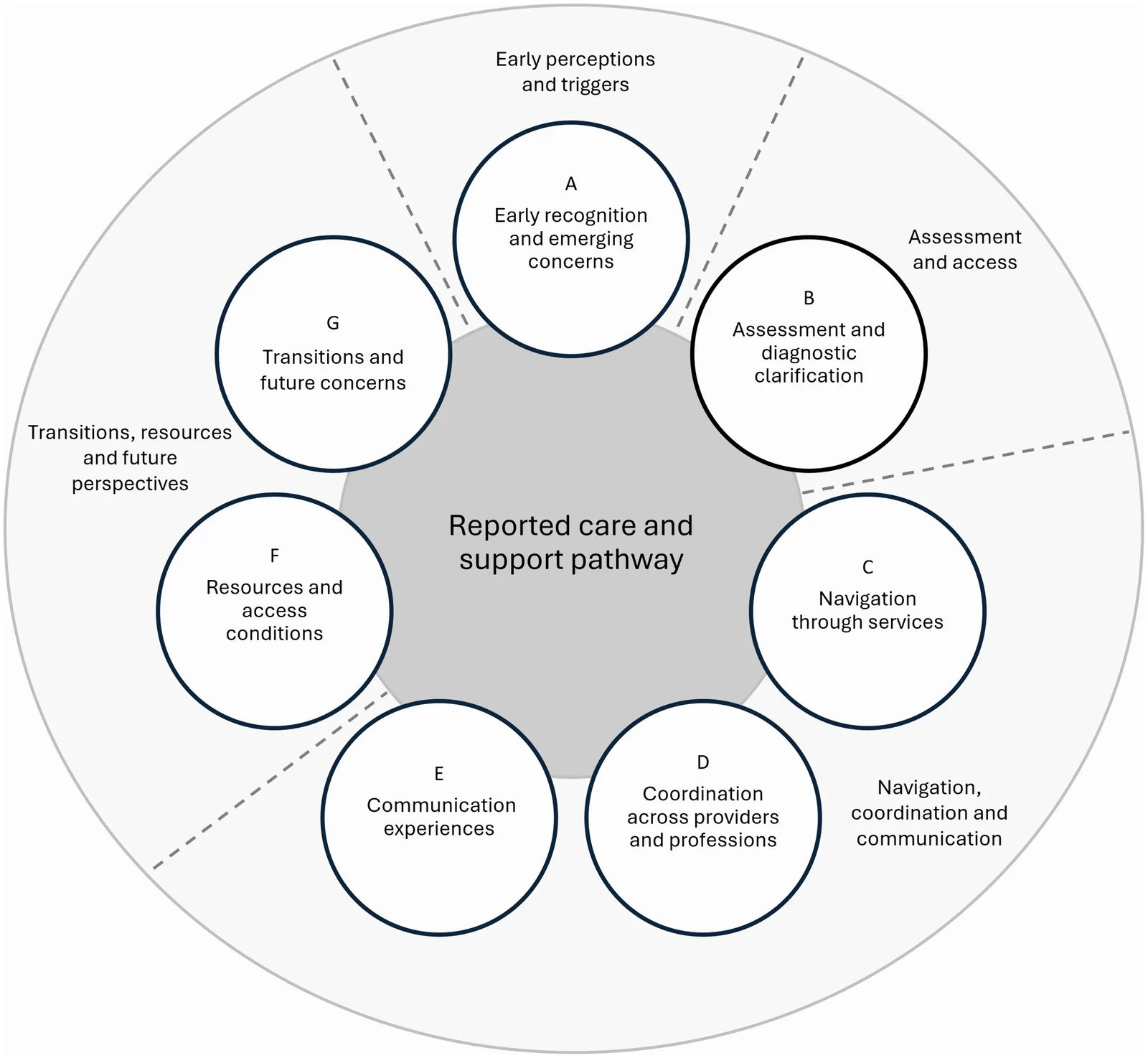
Reconstructed nodal points and process dynamics in the reported care and support pathway. The figure visualises recurring nodal points and decision situations (A–G) described by mothers along the reported care and support pathway. The pathway is not intended to represent a fixed linear sequence. Depending on the case, individual nodal points may occur in different orders, overlap, recur, or unfold partly in parallel. The four analytical units used in the Results section correspond broadly to these nodal points: early perceptions and triggers primarily relate to A; assessment and access primarily relate to B; navigation, coordination, and communication primarily relate to C, D, and E; and transitions, resources, and future perspectives primarily relate to F and G.

The interviews reconstruct recurring stages, nodal points, and decision situations along the care and support pathways reported by mothers. These are presented not as a fixed chronological sequence, but as recurring and partly overlapping process moments. The four overarching analytical units used below provide a reader-oriented structure for presenting the findings; they do not imply that all mothers moved through the same stages in the same order. Fig 1 visualises the reconstructed nodal points as a heuristic orientation framework.

Table 4 illustrates the four analytical units by means of selected direct quotations. It highlights different starting constellations at the beginning of the care pathway, the ambivalence of diagnostic clarification, the shifting of navigation and coordination work onto mothers, and the importance of resources and transitions for the further course of care. A comprehensive evidence table is provided in S4 File.

**Table 4:** Key findings and exemplary direct quotations for the four analytical units (short version).

Overarching analytical unit	Analytical focus	Exemplary direct quotation	Interview
Early perceptions and triggers of the care pathway	Early maternal perception as the starting point of the care pathway	“It became noticeable early on that his language was not developing like that of his siblings.”	I10
Early perceptions and triggers of the care pathway	Institutional feedback intensifies the pressure to act	“The first signs definitely came between the ages of 2 and 3 … and by age 3 we had already received the first feedback from the kindergarten that the child was not developing in an age-appropriate way, especially with regard to language.”	I3
Assessment and access as an ambivalent phase of orientation	Lack of diagnostic clarification creates burdensome uncertainty	“The problem, of course, is that it still hasn’t been diagnosed what kind of language disorder he actually has. So where does it come from? … the causes have not been clarified.”	I10
Assessment and access as an ambivalent phase of orientation	Diagnosis provides orientation and functional relief	“Over all these years, I’ve learned that with, let’s say, solid diagnoses, you can achieve much more than with just a description of the problems.”	I9
Navigation, coordination, and communication along the pathway	Lack of navigation shifts search and coordination work onto mothers	“You know, there isn’t really a contact person … you have to find everything out yourself and work everything out yourself.”	I6
Navigation, coordination, and communication along the pathway	Communication becomes burdensome when difficulties are framed as a parenting problem	“… being told that this is all a parenting issue, that burdens me so much, that people don’t acknowledge that this child is different, but always blame it on the mother’s parenting.”	I3
Transitions, resources, and future perspectives	Resources shape access and the further course of care	“It really makes a world of difference to know someone.”	I4
Transitions, resources, and future perspectives	Transitions and future perspectives remain partly burdened by uncertainty	“What is still burdensome, I think, is that we don’t know where this journey is going to lead.”	I10

*Note. The quotations were selected in line with the analytical logic of the Results section and represent, for each analytical unit, central patterns as well as one key contrast.*

### Early perceptions and triggers of the care pathway

In most interviews, the reported care and support pathway began with an initial perception that something was not developing as expected. The material suggests three typical starting constellations: an early maternal impression, institutionally triggered or intensified perceptions of a problem, and, in a smaller number of cases, first indications arising in medical or early childhood contexts. A single uniform starting point could therefore not be reconstructed. Rather, the perceived need for action emerged, depending on the case, from mothers’ own observations, feedback from others, or an interaction between both.

Several mothers described the beginning first and foremost as an early personal irritation or observation. One mother, for example, reported that it had become noticeable early on that her child’s language was not developing like that of his siblings (I10). In I3, too, the first signs were initially framed as a maternal perception before institutional feedback was added (“The first signs definitely came between the ages of 2 and 3, when we noticed …”; I3, first response block to the opening question).

At the same time, the beginning of the care pathway was repeatedly marked or significantly intensified by feedback from daycare or kindergarten. In I8, this became particularly clear when the mother stated that “that was really when it started in kindergarten” and described how “many people” told her that “something was wrong” with her child, even though she initially did not want to accept this because he appeared very different at home. In I3 as well, the perceived need for action was intensified by the first feedback from the kindergarten that the child was not developing in an age-appropriate way, especially with regard to language. In a few cases, first indications were also located in medical contexts, for example during a social paediatric assessment (“…at the SPZ … they said, ‘Well, language is somewhat noticeable’”; I6). By contrast, an explicit starting point via routine preventive check-ups was rarely reconstructed in the material; in I7, such an examination was mentioned more as a possible future occasion (“The U9 is scheduled for this autumn. Maybe it will be addressed then …”; I7).

Taken together, the interviews suggest that the beginning of the care and support pathway was not tied to a single, uniform point of origin. Rather, the perceived need for action arose from early personal observations, institutional signals, or the mutual reinforcement of both. These initial perceptions were usually followed by further phases of clarification and orientation.

### Assessment and access as an ambivalent phase of orientation

In the interviews, the first perceptions of concern were often followed by a phase of assessment and clarification that substantially shaped the further care pathway. However, this phase was not consistently experienced as clarifying. Rather, from mothers’ perspectives, diagnostic assessment appeared ambivalent: it could prolong uncertainty when diagnostic classification did not materialise at first or was postponed with reference to the child being still “too young”, while at the same time it could create orientation when naming or classifying the problem later on made the situation more tangible.

In several interviews, mothers described early attempts at clarification that initially did not result in a clear diagnostic classification. In I3, one mother reported that in an early assessment no diagnosis was made because it was considered “far too early” to do so (I3, section on early clarification/diagnostics). In the material, such situations do not appear neutral, but rather as a prolonged state of diagnostic uncertainty. Accordingly, another mother described the absence of a clear diagnostic classification as a continuing and burdensome source of uncertainty: “…it still has not been diagnosed what kind of language disorder he actually has” (I10, #00:14:19).

At the same time, the interviews also show that diagnostic labels could take on an important pragmatic function later in the process. Mothers described them not only as professional classifications, but also as prerequisites for further steps within the support system. This is particularly clear in I9, where the establishment of a diagnosis is retrospectively marked as relieving: “At last it had a name.” In the same context, the interviewee emphasised its functional relevance for gaining further access: “…with, let’s say, solid diagnoses, you get much further than with just a description of the problems” (I9, section on diagnosis/relief).

Overall, assessment in the interviews therefore appears not as a linear clarifying step, but as an ambivalent phase of orientation. It was experienced as burdensome above all when diagnostic classification was absent or postponed; it became relieving and practically relevant when, from mothers’ perspectives, diagnoses created tangibility, legitimacy, and access to further support and care services.

### Navigation, coordination, and communication along the pathway

As the care pathway progressed, mothers repeatedly reported that they themselves had to take on key tasks of navigation, coordination, and communication. In the material, this effort does not appear primarily as an expression of individual competence, but rather as a response to a care process that, from mothers’ perspectives, was characterised by opaque responsibilities, a lack of guidance, and fragile coordination between the services involved. This section therefore focuses on three closely interrelated aspects: the intensification of mothers’ own coordination work, fragile or contradictory interfaces between services and professions, and communication as a factor that could either relieve or burden. Orientation and relief emerged above all where responsibilities became comprehensible, contactability was ensured, and communication was experienced as understandable and respectful. Burden, by contrast, was reported primarily when processes were experienced as fragmented, contradictory, or devaluing.

First, several mothers described a marked intensification of self-organisation and searching activities within the support system. This concerned both appointment and application processes as well as the independent search for information and responsible services. In I9, the accumulation of demands became visible in what the mother described as “this diagnostic search, this running from doctor to doctor, from institution to institution.” In I6, the lack of clear navigation support was explicitly identified as a problem: “…there isn’t really a contact person … you have to find everything out yourself …” In I8, too, independent research appeared as a necessary part of navigation: “…I basically found it out myself through hours of searching on the internet.” What these accounts share is that coordination work appears not as a secondary accompanying task, but as a central maternal responsibility over the course of care.

Closely related to this, interfaces between the services and professions involved were described as fragile and, at times, contradictory. From mothers’ perspectives, information, priorities, and responsibilities often did not connect seamlessly. In I6, this became visible when the early intervention service concluded that the children also needed occupational therapy, the SPZ then “looked more closely” and confirmed this, but the mother still had to “argue” with the paediatrician, who stated: “Either speech therapy or occupational therapy,” not both at the same time. Overall, the interviews point to a need for reliable interface coordination that is frequently shifted onto families in everyday practice. This was also echoed in I7, where the mother stated that “the instances have nothing to do with each other,” apart from the speech therapist’s report requesting a follow-up prescription.

Within these constellations, communication emerged as a key operative factor. Interactions were described as supportive when they enabled accessibility, provided orientation, and took mothers’ concerns seriously; for example, one mother contrasted earlier experiences with a later service that she described as “significantly more empathic” and as continuing to advise and accompany the family (I9). In I3, the repeated framing of the issue as a matter of “parenting” was explicitly described as burdensome (“…that burdens me so much…”); in I8, the interviewee reported repeated external attributions suggesting that her child “…just needed to be properly brought up.” In I4, dismissive reactions in conversation were likewise described as “not respectful.”

Overall, the interviews show that navigation, coordination, and communication are closely intertwined over the course of care. Where orientation, responsibility, and comprehensibility were not systematically established, mothers increasingly took on coordinating and mediating functions. Where communication, by contrast, was experienced as accessible, clarifying, and respectful, it could have a relieving effect and contribute to mothers’ ability to act within the care pathway.

### Transitions, resources, and future perspectives

Over the further course of the reported care pathway, transitions and decision situations emerged as phases in which coordination demands intensified and resource-dependent differences in access became particularly visible. In these accounts, mothers described not only administrative shifts in responsibility, for example in the transition into the education system, but also conditions that shaped how effortful, rapid, or well-connected subsequent steps in the care pathway were experienced. Resources such as social networks, insurance status, and linguistic accessibility therefore appeared in the material as relevant contextual factors in managing the pathway.

Several mothers explicitly identified such resources as factors influencing access and the course of care. In particular, they referred to social contacts and prior knowledge, insurance status, and language and translation-related accessibility. In I4, the effect of social networks was summarised succinctly: “It really makes a world of difference to know someone.” In I5, one mother described private insurance status as an advantage in obtaining appointments, explaining that it placed them “in the fortunate position that no matter where we call, we get an appointment within the next two weeks.” Language and translation-related accessibility were also mentioned as relevant conditions of access; in I9, for example, the interviewee called for a low-threshold, multilingual overview “that could probably be translated quite easily into all kinds of languages.” These passages suggest that, from mothers’ perspectives, differences in access are shaped not only by the child’s needs, but also by the social, organisational, and linguistic resources available to the family.

Such conditions became particularly evident at transitions and points where responsibilities shifted. Above all, the transition into school was described in the interviews as a coordination-intensive and burden-prone phase. In I8, school life was experienced as repeatedly interrupted: “…my main activity at school was waiting for the school to call and say, ‘Please come and pick up your child.’” In the same interview, school support arrangements such as a pooled support model, in which support is shared across several children rather than assigned exclusively to one child, were described as problematic because changing reference persons and substitute arrangements were experienced by the mother as generating additional instability. Beyond concrete transition situations, the further course of development was also described in some interviews as open and uncertain. Thus, in I10, uncertainty about the future pathway was explicitly described as burdensome: “What is still burdensome, I think, is that we don’t know where this journey is going to lead.”

Overall, the interviews show that transitions do not merely represent administrative thresholds, but rather phases of intensified demands in which questions of responsibility, continuity, and future planning become particularly acute. At the same time, the resources available to families shape how such phases can be managed. Resources appear both as levers for quicker or easier access and, in their absence, as barriers or factors that increase effort. Transitions and future perspectives therefore mark an area in which the structural demands of the care system and unequal starting conditions become especially intertwined from mothers’ perspectives.

### Integrative synthesis

Overall, the reported care and support pathway appears not as a linear trajectory, but as a sequence of recurring situations of perception, assessment, coordination, and transition in which demands for orientation, responsibility, comprehensibility, and capacity for action repeatedly arise. With regard to the first research question, mothers described burden and relief as closely linked to how understandable, responsive, and connected the care system became at relevant points along the pathway. With regard to the second research question, navigation, coordination, and decision-making tasks emerged as substantial work that mothers often had to take on themselves, especially where responsibilities were unclear or interfaces were weakly coordinated. With regard to the third research question, communication and collaboration at interfaces appeared as central mechanisms through which orientation was either strengthened or undermined. With regard to the fourth research question, the material points to support needs related above all to reliable orientation, clearer responsibilities, accessible information, respectful communication, and better coordination across institutional transitions. Explicit emotional burden and relief were considered only where they were explicitly articulated in the material.

## Discussion

### Key findings

This qualitative study reconstructs mothers’ perspectives on the care and support pathway of children with speech, communication, and developmental concerns as a non-linear process shaped by multiple institutional interfaces. The interviews do not portray the reported pathway as a straightforward sequence of clearly defined steps. Rather, they suggest a recurring series of situations involving perception, clarification, coordination, and transition, in which demands for orientation, responsibility, and capacity for action repeatedly arise.

What becomes particularly clear is that mothers often do not merely accompany this process, but must themselves structure, coordinate, and hold it together to a considerable extent. In the data, navigation and coordination work appear not primarily as voluntarily assumed additional tasks, but as responses to opaque responsibilities, limited coordination between the services involved, and the absence of reliable guidance. Burden arises especially where diagnostic clarification is lacking, responsibilities remain unclear, transitions generate additional uncertainty, or communication is experienced as dismissive, devaluing, or insufficiently responsive. Relief, by contrast, is described particularly where mothers’ observations are taken up, problems are interpreted in a comprehensible way, next steps are made understandable, and support is experienced as accessible and appropriate.

The findings thus point to care and support pathways in which the central issues concern not only the availability of individual services, but also the production of orientation, comprehensibility, continuity, and connectedness over time. Communication emerges here not as a mere accompanying factor, but as a central process dimension through which orientation, validation, and capacity for action are either enabled or impeded.

### Interpreting non-linear care as a process of navigation and coordination

The findings suggest that the reported care and support pathway is best understood, first and foremost, as a complex process of navigation and coordination. Previous work has described the diagnostic trajectory of families of children with developmental disabilities in very similar terms: from parents’ perspectives, it begins not with the formal evaluation itself, but already with first developmental concerns, and extends across multiple stages of access, referral, and assessment; barriers to accessibility and continuity are identified as particularly problematic in this process [3]. A comparable process logic is also evident in Canadian stakeholder research on diagnostic services for young children with developmental disabilities, in which families often experienced the care and assessment trajectory not as a coherent sequence, but as a fragmented pathway between different services that remained difficult to navigate in the absence of formalised procedures and reliable navigation support [2]. This is further echoed in qualitative findings from an urban, resource-limited care context, in which caregivers likewise described access to services as a frustrating process of navigating a fragmented system, while professionals viewed their ability to support families as constrained by lack of time and limited knowledge of available services [21].

In the present material as well, care does not appear, from mothers’ perspectives, primarily as a coherent system with clear responsibilities, but rather as an arrangement of different institutions, professions, and jurisdictions between which information, decisions, and next steps do not always connect seamlessly. Burden arises here not only from the child’s support needs, but also, importantly, from the demands that a care process shaped by multiple interfaces places on families. Similarly, previous work has described burden as a consequence of a care system in which waiting times, inconsistent procedures, repeated informational effort, and insufficient coordination between services are effectively shifted onto families [2]. Related work likewise reports burden particularly where families had to manage waiting times, a lack of support in interim phases, repeated informational effort, and disruptions in continuity of care [3].

The interviews therefore suggest that mothers’ work along the care and support pathway increases above all where systemic coordination, continuity, or transparent responsibilities are not sufficiently established. At interfaces in particular, such as those between daycare, medical assessment, therapy, early intervention, and school, situations repeatedly arise in which mothers themselves must mediate, follow up, explain, organise, and prioritise. In doing so, they take on tasks that the system does not reliably assume on their behalf. Transitions, especially into school, appear as particularly condensed phases in which uncertainty, coordination effort, and future-related concerns intensify once again.

From this perspective, the findings can be read as pointing to problems of fit between support needs and the organisation of care. What is decisive is not merely whether services exist, but how connected, understandable, and coordinable they actually become in the everyday lives of families. The contribution of the present study therefore lies less in describing individual deficits in provision than in reconstructing an underlying process logic: from mothers’ perspectives, care repeatedly becomes fragile at precisely those points where orientation, continuity, and responsibility are not reliably established. Conceptually, this is also consistent with the argument that family-oriented services should not end with child-focused interventions, but should also include support, information, and coordination for parents and families themselves [22].

### Relational and organisational health literacy as an interpretive framework

For interpreting these findings, health literacy proves to be a useful analytical framework, provided that it is understood relationally. From this perspective, health literacy does not reside solely in the individual abilities of parents, but emerges through the interplay between family-related preconditions and the demands of care systems, organisations, and specific communicative situations. This is particularly relevant for the present findings because, in child care contexts, parents and other caregivers commonly act on behalf of the child, integrate information from different contexts, and make decisions under conditions in which responsibilities and next steps are not always clearly discernible.

The findings therefore speak against an individualising deficit perspective on families. In the material, the fact that families search for information, coordinate appointments, clarify responsibilities, or work through contradictions between different recommendations does not appear as an expression of insufficient competence, but as a consequence of a system that does not consistently provide orientation, comprehensibility, and continuity itself. Against this background, health literacy should be understood not only as the ability to find, understand, appraise, and apply information, but also as a question of how the care system structures its demands.

Closely linked to this is the notion of organisational health literacy. From mothers’ perspectives, high levels of burden arise particularly where information, procedures, and responsibilities are not designed in ways that make them understandable and manageable even without specialised system knowledge or informal resources. This is consistent with work on organisational health literacy in which health literacy responsiveness is understood as a context- and setting-related responsibility, with communication, access and navigation, user involvement, and checking understanding described as central fields of action [15,23]. The findings can therefore be read as indicating that health literacy-sensitive care in the present context must encompass not only audience-appropriate information, but also structural orientation, comprehensible process management, and reliable clarification of responsibilities. In this way, the perspective shifts from asking whether parents navigate “well enough” to asking how care can be organised so that navigation is not disproportionately shifted onto families. The fact that access and navigation are counted among the central criteria of health-literate organisations further reinforces this perspective [15].

### Communication as a mechanism of orientation, validation, and capacity to act

The findings make clear that communication plays a key role along the reported care and support pathway. This is consistent with systematic reviews on family-centred care, in which family-provider partnership is conceptualised as a functional core of family-centred care and linked to better communication and greater satisfaction [7]. Work on the provider-family relationship likewise explicitly treats communication and relationship quality as part of care quality; qualitative studies have identified transparency, communicative openness, availability, trust, and mutual respect as particularly important features in this regard [2,19].

In the present material, mothers experienced interactions as supportive particularly when their observations were taken seriously, uncertainties were interpreted in a comprehensible way, and next steps were made transparent. In such cases, communication contributed to restoring or strengthening mothers’ capacity to act: it reduced uncertainty, created tangibility, and made the further course of care at least provisionally understandable. This is also in line with reviews in speech-language therapy, in which acknowledging the person, responding sensitively, and involving caregivers are described as central processes in relationship building [24].

By contrast, communication was experienced as burdensome when concerns were trivialised, difficulties were framed primarily as matters of parenting, or mothers’ observations were not treated as legitimate perceptions. Comparable patterns have also been described in studies of diagnostic trajectories: parents often reported feeling unheard, especially before formal diagnosis, experienced professionals as cold or mechanical, and criticised a lack of transparency in communication; conversely, empathy, listening, openness, and personal support were described as key facilitating factors [3]. In autism-specific diagnostic contexts as well, passive or dismissive responses have been linked to delays in the diagnostic process [8]. In the present material, such interactions appear not merely as unpleasant, but as moments in which orientation is lost, responsibility is implicitly shifted back onto the family, and experiences of shame or uncertainty may arise.

The data do not measure empathy in a narrow sense as an independent variable. They do, however, clearly show that mothers experience not only factual information, but also communication that is understanding, respectful, and validating as highly relevant. In this sense, the findings are compatible with concepts of empathic and health literacy-sensitive communication: empathy appears here less as an additional “soft” quality alongside information than as a communicative precondition for information to become orienting, trust-building, and relevant for action in the first place.

### The ambivalent role of diagnosis and assessment

One of the study’s central findings concerns the ambivalent role of diagnosis and assessment. The fact that families experience the diagnostic phase as both necessary and burdensome is also evident in previous work on diagnostic trajectories [3]. There, the period before formal diagnosis and the transitions following diagnosis were described as particularly problematic, whereas the assessment phase itself tended to be experienced as relieving when it was conducted in a timely, transparent, and relationship-oriented manner [3].

In the present material as well, earlier attempts at clarification were not consistently described as clarifying. Diagnostic classification could initially fail to materialise, be postponed, or provide only limited orientation for mothers. Comparable patterns have also been described for autism-specific diagnostic pathways: early signs were sometimes interpreted as normal variation or relativised with the suggestion that it was still “too early,” while the diagnostic process itself could become burdensomely prolonged through long waiting times and delayed access to services [8]. In such situations, uncertainty was not experienced in the present material as neutral, but often as a burdensome suspended state in which neither the nature of the problem nor the next steps became sufficiently tangible.

At the same time, the data show that diagnostic labels could assume an important pragmatic function later in the process. Mothers experienced them not only as professional classifications, but also as socially and organisationally effective points of orientation that could facilitate access, structure options for action, and make the problem more communicable to others. In this sense, diagnosis appears in the present material neither simply as a solution nor exclusively as a problem, but as an ambivalent nodal point within the care and support pathway: it can create insecurity when absent or unclear, while also becoming relieving when it generates tangibility, legitimacy, and continuity.

For interpreting these findings, it is therefore important to understand diagnosis not merely as a medical act, but also as a communicatively and organisationally effective process. From mothers’ perspectives, what appears decisive is not only that some form of classification is reached, but whether that classification is conveyed in a way that is understandable and actually makes the next steps manageable.

### Practical implications and functional requirements

The implications of the present study are best understood as functional requirements derived from mothers’ reconstructions of care pathways, not as evidence for the effectiveness of specific intervention or service models. The interview material points to four interrelated requirements: reliable orientation and navigation support, understandable and responsive communication, better coordination across interfaces, and equity-sensitive access.

First, the findings point to the importance of reliable orientation and navigation support. This requirement is grounded in accounts in which mothers described having to search for responsible services, organise appointments, interpret procedures, and work out next steps largely on their own. The need for orientation was particularly visible at diagnostic turning points, in situations of unclear responsibility, and during transitions. In such situations, families need not only information about individual services, but also support in understanding which steps are relevant when, who is responsible, and how different services are connected. This data-derived requirement is consistent with previous work highlighting the relevance of case navigation, formalised procedures, information transfer, and post-diagnostic guidance in developmental care pathways [2,3,21].

Second, the results underline the importance of understandable, structured, and responsive communication. In the interviews, communication was experienced as relieving when mothers’ observations were taken seriously, uncertainty was explained in a comprehensible way, and concrete next steps became more manageable. Conversely, communication became burdensome when concerns were trivialised, difficulties were attributed primarily to parenting, or mothers felt dismissed or blamed. From these accounts, health literacy-sensitive communication in this context means more than plain language in a narrow sense. It includes transparency, checks of understanding, validation of mothers’ observations, and interactional practices that help families translate information into feasible action. This is consistent with criteria of organisational health literacy, in which communication, access and navigation, user orientation, and checking understanding are described as central organisational tasks [15].

Third, the interviews indicate a need for improved coordination across institutional and professional interfaces. This implication follows from accounts in which responsibilities between paediatric care, therapy, early intervention, daycare, school, and other services were experienced as unclear, contradictory, or weakly connected. Where coordination between the professions and institutions involved was lacking, coordination work was shifted to a considerable extent onto mothers and, by extension, their families. Practical consequences therefore concern, in particular, clearer responsibilities, comprehensible handovers, and more binding coordination processes between medical, therapeutic, early childhood educational, and school contexts.

Fourth, the findings suggest that access and burden are shaped by unequal resource conditions. In the material, social networks, prior knowledge, insurance status, linguistic accessibility, and knowledge of the system influenced how quickly, smoothly, and effectively support could be reached. This does not allow conclusions about the distribution of such resources in the wider population, but it indicates that care pathways may become more burdensome when access tacitly depends on informal knowledge or high levels of individual initiative. Other studies likewise show that progress along diagnostic pathways depends not only on the child’s needs, but also on the family’s possibilities for action [3]. For families with limited language proficiency, professional interpretation may be an important precondition for understandable care [17].

Taken together, these implications should be read cautiously. The study does not test whether specific navigation, counselling, or coordination models are effective. It does, however, identify functional requirements that were repeatedly relevant in mothers’ accounts: orientation that makes next steps visible, communication that is understandable and validating, responsibilities that are coordinated across interfaces, and access pathways that do not depend disproportionately on individual resources.

## Limitations

The findings should be understood as subjective reconstructions of mothers’ experiences. The aim of the study was not to provide an objective evaluation of care quality or to determine frequencies, but rather to reconstruct meanings, process-related demands, and conditions associated with burden and relief along the reported care and support pathway.

An important limitation concerns the sample and the access pathways through which participants were recruited. The interview sample consisted of mothers whose families had entered or recently used occupational therapy and/or speech and language therapy services. The findings therefore do not represent families who never accessed such services, disengaged at an early stage, or were not reached through the recruitment pathways used in this study. Consequently, access-related findings should be understood as referring to mothers who had already reached parts of the support system despite existing barriers. It is plausible that orientation, access, and coordination barriers may be at least as relevant for families not reached by this study; however, this remains an inference beyond the present data and cannot be examined empirically here.

Although the study protocol primarily targeted parents of preschool-aged children, the open age question in the survey resulted in a realised sample comprising mothers of children across school age and adolescence. The findings should therefore not be interpreted as age-specific statements, but rather as a reconstruction of recurring process-related demands across different phases and contexts. The interviews were also retrospective accounts; recall periods varied, and care structures may have changed over time. These accounts are therefore best understood as subjective reconstructions of experienced care pathways, not as direct contemporaneous observations of service provision.

Interview duration and narrative depth also varied across cases. Shorter interviews were retained when they contributed relevant process-related information to the cross-case analysis; however, the findings should not be read as resting on equal narrative depth across all interviews.

Further limitations concern self-selection, diagnostic heterogeneity, and the composition of the realised interview sample. Diagnoses and developmental concerns were based on mothers’ reports and were not clinically verified. The heterogeneity of the children’s reported concerns limits disorder-specific conclusions, but is compatible with the study’s focus on cross-cutting navigation, coordination, and communication demands along care pathways. The realised interview sample consisted exclusively of mothers; the findings therefore reflect maternal perspectives and do not capture the full range of possible experiences among fathers, other caregivers, or parents as a broader group. Accordingly, the navigation and coordination work reconstructed in this study should be interpreted specifically as described from mothers’ perspectives rather than as representative of parental caregiving more broadly. The study was not designed to compare gendered caregiver roles, and such comparisons remain beyond its scope.

The quality assurance of coding must also be interpreted with caution. The reported intercoder comparison was based on a subset of the material and was calculated as percentage agreement. This figure should therefore be understood as an indicative measure of coding consistency achieved within the project, not as a measure of strictly independent reliability. At the same time, the documented category system, the case-by-category matrix, the linked supporting excerpts, and the supplementary materials strengthen the traceability of the analytic process.

## Conclusion

Mothers do not reconstruct care and support pathways relating to their children’s speech, communication, and developmental concerns as linear routes, but as complex processes of navigation and coordination within a system marked by multiple interfaces. From mothers’ perspectives, critical points arise especially where orientation, diagnostic classification, communication, responsibilities, and transitions are not designed in ways that are sufficiently understandable, coordinated, and connected. Support structures should therefore not merely provide additional individual services, but above all foster reliable orientation, coordinated responsibilities, and communication that not only informs, but is also experienced by mothers as understanding, respectful, and responsive.

## Supporting information

S1 File Full codebook. Complete codebook used for the qualitative content analysis, including main categories, subcategories, definitions, and anchor examples.
(DOCX)

S2 File Case-by-category matrix. De-identified case-by-category matrix documenting coded main categories across interviews together with supporting excerpts used in the cross-case analysis.
(DOCX)

S3 File Interview materials. Interview guide and chronology table used in the qualitative interview study, together with a brief note on the development of the supporting interview aid from the pilot phase to the main study.
(DOCX)

S4 File Comprehensive evidence table. Comprehensive evidence table containing the interview excerpts and closely condensed evidence passages underlying the main analytic claims and the four overarching analytical units presented in the Results section.
(DOCX)

S5 File COREQ checklist. Consolidated criteria for reporting qualitative research (32-item checklist) completed for the present qualitative interview study.
(DOCX)
