# Supplementary material for: Mothers’ experiences of care pathways for children with speech and developmental concerns in Germany: A qualitative study of navigation, transitions, and communication
Source: PLoS One. 2026 Sep 16;21(9):e0357294. doi: 10.1371/journal.pone.0357294 (PMC13580943; doi:10.1371/journal.pone.0357294)
Supplement: Supplementary file 1 [file pone.0357294.s001.docx]

**S1 File. Full codebook.**

Complete codebook used for the qualitative content analysis, including main categories, subcategories, levels, definitions/memos, and selected anchor examples.

| Main category | Subcategory/path | Level | Definition/memo | Selected anchor example |
| --- | --- | --- | --- | --- |
| Professionals/institutions |  | 1 | All professionals, services, and institutions mentioned along the care pathway. | — |
| Professionals/institutions | School | 2 | Statements about the role and suitability of schools (mainstream, special, or private schools), particularly with regard to support, class size, and inclusion. | “At some point, my main task at school was to wait for the school to call and say: ‘Come and pick up your child.’” (I8) |
| Professionals/institutions | Occupational therapy | 2 | References to occupational therapy support in the context of developmental language disorder or psychosocial concerns. | “Either speech therapy or occupational therapy.” (I6) |
| Professionals/institutions | Social paediatric centre (SPZ) | 2 | Specific statements about the social paediatric centre, including accessibility, diagnostic assessment, counselling, and its role in the process. | “...at the SPZ ... they said, ‘Well, language is somewhat noticeable’.” (I6) |
| Professionals/institutions | Early intervention services | 2 | Experiences with early intervention services (home-based or centre-based), including organisational aspects, satisfaction, and access. | “As the first point of contact, we were advised to go to an early intervention centre. They were very nice colleagues who came into the kindergarten and looked at my child.” (I8) |
| Professionals/institutions | Paediatrician | 2 | Statements about contact with paediatricians, regardless of evaluation. Used to capture the landscape of actors involved. | CA: the paediatrician was described as relevant to decisions about further therapy and prescriptions. (I6) |
| Professionals/institutions | Speech and language therapy | 2 | Direct statements about speech and language therapy, regardless of evaluation. May be differentiated further by positive or negative valence where relevant. | At that time they didn’t want to diagnose anything and said it was far too early. Recommendation for speech and language therapy emerged only later. (I3) |
| Professionals/institutions | Private school as an option | 2 | Mention of private school as a possible solution in the context of insufficient support structures in the public education system. | CA: A smaller private school was considered as a possible option because of smaller class sizes. (I3) |
| Professionals/institutions | Other professional | 2 | Unspecified or less frequently mentioned professionals involved in the process (e.g., counselling services, external therapists). | CA: An external professional maintained close communication with early educational settings. (I4) |
| Professionals/institutions | Psychomotor therapy/support | 2 | References to accompanying motor-based support measures in the context of therapy or daycare. | — |
|  |  |  |  |  |
| Emotional well-being |  | 1 | Mothers’ subjective emotional responses to the care situation. | — |
| Emotional well-being | Emotional positive | 2 | Relief, reassurance, hope, optimism, or a sense of increased security. | “At last it had a name.” (I9) |
| Emotional well-being | Emotional negative | 2 | Burden, uncertainty, worry, frustration, or overwhelm. | “What is still burdensome, I think, is that we don’t know where this journey is going to lead.” (I10) |
| Emotional well-being | Future concerns | 2 | Uncertainty regarding the child’s educational, occupational, or social future. | “What is still burdensome, I think, is that we don’t know where this journey is going to lead.” (I10) |
|  |  |  |  |  |
| Interdisciplinary collaboration |  | 1 | Experiences with coordination between different professional groups. | — |
| Interdisciplinary collaboration | Successful | 2 | Positive experiences of collaboration, for example as coordinated or effective. | “She was always in close exchange with the kindergarten and the crèche. And that worked quite well.” (I4) |
| Interdisciplinary collaboration | Negative | 2 | Lack of coordination, contradictory statements, or discontinuities within the system. | “The instances have nothing to do with each other.” (I7) |
|  |  |  |  |  |
| Resources |  | 1 | Subjectively experienced sources of relief or burden in everyday family life. | — |
| Resources | Positive | 2 | Family-related, social, or personal strengths experienced as helpful. | “It really makes a world of difference to know someone.” (I4) |
| Resources | Social support (family) | 2 | Relief provided by family members, for example grandparents or a partner. | — |
| Resources | Negative | 2 | Lack of resources, for example time, money, childcare, or language skills. | CA: language and translation-related accessibility were described as relevant to navigating care. (I9) |
|  |  |  |  |  |
| Organisational barriers/critique |  | 1 | Structural challenges and points of criticism within the system. | — |
| Organisational barriers/critique | Education system | 2 | Criticism relating to schools, inclusion, class sizes, and related issues. | CA: Changes or discontinuities in school support arrangements were experienced as burdensome. (I8) |
| Organisational barriers/critique | Parental role responsibility | 2 | Mothers experience themselves as being forced to take on a coordinating role. | “You know, there is no single contact person ... you have to find everything out yourself and work everything out for yourself.” (I6) |
| Organisational barriers/critique | Changing professionals | 2 | Frequent staff changes and lack of continuity. | — |
| Organisational barriers/critique | System responsibility | 2 | Expectation that support should be initiated and organised by the system. | CA: need for clearer support and guidance in navigating the system. (I3) |
| Organisational barriers/critique | Support system lacks clarity | 2 | Lack of clarity and structure within the support and care system. | “You know, there is no single contact person ... you have to find everything out yourself and work everything out for yourself.” (I6) |
| Organisational barriers/critique | Waiting times/bureaucracy | 2 | Long waiting times, registration procedures, and administrative barriers. | CA: prolonged diagnostic search and repeated movement between services were described as burdensome. (I9) |
|  |  |  |  |  |
| Communication |  | 1 | Experiences of communication with professionals. | — |
| Communication | Positive | 2 | Helpful, empathic, and clear communication. | CA: later service experiences were described as significantly more empathic and as providing ongoing guidance. (I9) |
| Communication | Negative | 2 | Unclear, contradictory, or delayed communication. | “...being told that this is all just a matter of parenting burdens me so much...” (I3) |
|  |  |  |  |  |
| Contextual conditions |  | 1 | Structural conditions influencing care and access. | — |
| Contextual conditions | Favourable | 2 | Structural aspects that function smoothly and facilitate care. | “That is why we are in the fortunate situation that wherever we call, we get an appointment within the next two weeks.” (I5) |
| Contextual conditions | Unfavourable | 2 | Hindering conditions such as staff shortages or waiting times. | CA: Service disruptions and everyday organisational demands affected the pathway. (I10) |
|  |  |  |  |  |
| Wishes |  | 1 | Mothers’ explicit wishes for improving the system. | — |
| Wishes | Fixed contact person | 2 | Wish for continuous guidance by one consistent contact person. | CA: need for an overall plan and clearer ongoing guidance. (I6) |
| Wishes | Information needs | 2 | Wish for more information about the condition, its course, and related issues. | “If there were some kind of guide for all these questions, it could probably be translated quite easily into all sorts of languages.” (I9) |
| Wishes | Parent exchange | 2 | Wish for exchange with other parents. | — |
|  |  |  |  |  |
| Clinical picture |  | 1 | Characteristics of the problem situation and the diagnostic process. | — |
| Clinical picture | Unclear condition | 2 | Unclear diagnosis despite attempts at clarification. | “The problem, of course, is that it still hasn’t been diagnosed what kind of language disorder he actually has. So where does it come from? ... the causes have not been clarified.” (I10) |
| Clinical picture | Complex problem situation | 2 | Multiple interrelated problems or combined areas of difficulty. | CA: complex developmental and school-related difficulties structured the pathway. (I8) |
| Clinical picture | Stigmatisation / inclusion concerns | 2 | Concerns about social exclusion or inclusion-related disadvantages. | CA: School support arrangements raised concerns about continuity and inclusion. (I8) |
| Clinical picture | Early signs noticed | 2 | Early indications of developmental delay or atypical development. | “It became noticeable early on that his language was not developing like that of his siblings.” (I10) |
| Clinical picture | Diagnostic process | 2 | Statements about the process of diagnostic assessment. | “At that time they didn’t want to diagnose anything and said it was far too early.” (I3) |
| Clinical picture | Trust in professionals | 2 | Expressions of trust in professionals. | “The fact that the doctor trusted me so much ... that was already a basis of trust.” (I7) |
| Clinical picture | Recognition and gratitude | 2 | Expressions of appreciation for professionals. | “I am very grateful that it runs so straightforwardly.” (I7) |

**Note.** Anchor examples are provided to illustrate the meaning of categories and subcategories. Where concise direct quotations were not suitable, contextual anchors (CA) were used. For readability, anchor examples in this codebook are reported with interview ID only; more detailed evidence is provided in S2 and S4. Direct identifiers were removed, and potentially identifying contextual details were generalised or omitted where this did not affect the substantive meaning.
