# Supplementary material for: Mothers’ experiences of care pathways for children with speech and developmental concerns in Germany: A qualitative study of navigation, transitions, and communication
Source: PLoS One. 2026 Sep 16;21(9):e0357294. doi: 10.1371/journal.pone.0357294 (PMC13580943; doi:10.1371/journal.pone.0357294)
Supplement: Supplementary file 2 [file pone.0357294.s002.docx]

**S2 File. Case-by-category matrix.
Legend.** The columns Professionals/institutions, Contextual conditions, Clinical picture, and Wishes serve primarily as contextual descriptors. The remaining columns represent coded main categories based on the codebook provided in S1 File. Up to three supporting excerpts are listed per cell. Where available, entries include the interview ID and time stamp as documented in the transcript. Where no concise direct quotation was suitable, a brief contextual anchor is provided. Empty cells indicate that no clearly assignable supporting excerpt was identified in the available transcripts.
All interview data presented in this matrix were de-identified for publication. Names of persons, places, and institutions were removed or generalised, and potentially identifying contextual details were further generalised where this did not affect the substantive meaning.
**Note on quotations.** All direct quotations were translated from German into English by the authors. CA denotes a contextual anchor, that is, a brief analytic paraphrase used for documentation within the matrix; CA entries do not represent verbatim quotations.

| Interview | Emotional well-being | Professionals/institutions | Interdisciplinary collaboration | Communication | Organisational barriers/critique | Contextual conditions | Resources | Clinical picture | Wishes |
| --- | --- | --- | --- | --- | --- | --- | --- | --- | --- |
| I3 | 00:15:41, “…that burdens me so much…” in response to parenting-related attribution. | CA: speech and language therapy was recommended only later; early educational settings were a key institutional context. | CA: coordination demands became visible across early educational feedback, delayed diagnostic clarification, and later educational decisions. | 00:15:41, difficulties framed as “a matter of parenting.” | CA: diagnostic clarification was initially considered too early, contributing to delayed access to diagnosis and support. | CA: challenges surrounding an educational transition shaped the pathway. | CA: family and contextual resources intersected with concerns raised in early educational settings. | CA: first signs were noticed in early childhood; no diagnosis was made initially because clarification was considered too early. | CA: need for clearer support and guidance in navigating the system. |
| I4 | CA: long-standing therapy need from very early childhood; relief associated with access to supportive contacts. | CA: therapy need was apparent from very early childhood; support involved several services over time. | CA: coordination across services and settings remained relevant over an extended pathway. | 01:05:15–01:05:45, platitudes and minimisation were experienced as “not respectful”; 01:04:54, “Just take it seriously.” | CA: the mother had to organise support largely herself across a long pathway. | CA: the care pathway was shaped by early and ongoing support needs. | CA: personal contacts were described as an important resource in accessing support. | CA: early and persistent developmental concerns requiring therapy. | 01:04:54, wish to be taken seriously and not met with platitudes. |
| I5 | CA: self-doubt and emotional strain were indicated in relation to early concerns and help-seeking. | CA: multiple service contacts across the broader care history. | CA: parallel service use was possible and comparatively accessible. | CA: service contacts were experienced as responsive in at least some situations. | CA: fewer structural barriers were described, partly linked to available access-related resources. | CA: an educational transition formed part of the broader pathway. | CA: insurance-related resources facilitated access to appointments. | CA: concerns were addressed early and proactively. | — |
| I6 | CA: considerable everyday strain; future-related concern remained salient. | CA: access to support depended strongly on parental initiative and coordination across services. | CA: therapy options were experienced as constrained rather than coordinated. | CA: uncertainty about the overall plan and support structure. | CA: lack of a stable contact person contributed to parents having to navigate support largely on their own. | CA: complex family care responsibilities and formal assessments shaped the pathway. | CA: language proficiency facilitated system navigation. | 00:04:59, initially no concerns were noted; later signs prompted renewed clarification. | 00:17:13, need for an overall plan and clearer ongoing guidance. |
| I7 | CA: little explicit emotional burden was articulated; prior professional or system-related knowledge buffered uncertainty. | CA: paediatric care and routine paediatric assessment were the main institutional points of reference. | CA: no major interprofessional coordination issues were foregrounded. | CA: no major communication conflicts were described. | CA: the case suggests that navigation may be more difficult without prior professional or system-related knowledge. | CA: prior professional knowledge shaped interpretation and timing of help-seeking. | CA: prior professional knowledge functioned as a resource. | CA: early signs were noticed by the mother; routine paediatric assessment was mentioned as a possible future trigger rather than an initial starting point. | — |
| I8 | CA: school-related strain and emotional burden were described. | CA: educational and support services formed key institutional interfaces. | CA: changes or discontinuities in school support arrangements shaped coordination needs. | CA: difficulties were repeatedly framed in parenting terms; recommendations for “more strictness” were reported. | CA: recurrent disruptions in school attendance and support were described. | CA: changes in school context and repeated school-related disruptions shaped the pathway. | CA: need for guidance and orientation remained high; language-related resources were not central. | CA: complex developmental and school-related difficulties structured the pathway. | CA: wish for more stable and individualised support arrangements. |
| I9 | “At last it had a name.”; CA: diagnosis brought relief and orientation. | CA: multiple health, therapeutic, and educational services formed part of a long support pathway. | CA: coordination across multiple service settings remained relevant over time. | CA: some explanations initially remained hard to understand; later re-interpretation became possible. | CA: access to support was shaped by thresholds, classifications, and institutional rules. | CA: institutional thresholds and changing support arrangements shaped the pathway. | CA: language access and accessible information were relevant cross-cutting issues. | CA: diagnostic clarification provided orientation and legitimacy. | CA: wish for low-threshold, accessible guidance materials. |
| I10 | 00:12:25, “What is still burdensome, I think, is that we don’t know where this journey is going to lead.” | CA: health, educational, and therapeutic services were involved. | CA: support was present, but did not resolve the central lack of diagnostic orientation. | CA: communication did not resolve the central uncertainty surrounding diagnosis and next steps. | 00:14:19, missing diagnosis/classification remained an ongoing problem. | CA: everyday organisational demands affected the pathway. | CA: broader resource dependence remained only partly explicit. | 00:14:19, unresolved question of what kind of language disorder was present; broader developmental concerns were described. | CA: desire for clearer diagnostic orientation and next-step guidance. |
