# Supplementary material for: Mothers’ experiences of care pathways for children with speech and developmental concerns in Germany: A qualitative study of navigation, transitions, and communication
Source: PLoS One. 2026 Sep 16;21(9):e0357294. doi: 10.1371/journal.pone.0357294 (PMC13580943; doi:10.1371/journal.pone.0357294)
Supplement: Supplementary file 3 [file pone.0357294.s003.docx]

**S3 File. Interview materials.**

This supporting file contains the interview guide used in the qualitative interview study, the chronology table sent to participants before the interview, and a brief note on the development of the supporting interview aid from the pilot phase to the main study.

**Note.** The interviews were originally conducted in German. The interview guide and supporting materials reproduced here were translated into English by the authors for this supplement. Interview guide for the main study.

**Interview structure**

1. Welcome and thanks for participation
2. Introduction by the interviewer (standardised script; verbatim or paraphrased)
3. Interview (chronologically along the care pathway; clarification and probing questions)
4. Closing (thanks and opportunity for final questions)

**Standardised introduction (script)**

“Different institutions and professional groups are involved in the care of children in Germany, for example educational settings such as schools or kindergartens, medical providers such as paediatricians, or therapists (speech and language therapy, occupational therapy, psychology, etc.).”

“In our research, we would like to better understand how parents experience educational and health-related care and the interaction between these areas, including everyday educational settings, support in daycare or school, paediatric care, therapeutic services, information, and communication with parents.”

“You can help us by describing where you encountered obstacles or problems and what you experienced as helpful and supportive along your child’s care pathway. We would therefore like to ask you to tell us your story. We sent you a table in advance. Do you have it with you? If so, you can use it as a memory aid. If it is not available, the interview can still proceed without it.”

“I will guide you through the interview and record our conversation. I would like to emphasise once again that we will treat your personal data confidentially and that no conclusions can be drawn about you or your family. After the recording, our conversation will be transcribed and names will be changed. The video file will be deleted.”

**Interview opening and main prompt**

“Okay, then we can begin, and I am now starting the recording.”

“I would like to trace your pathway as chronologically as possible. The best place to start is to tell me when you, or someone else, first noticed something about your child …”

**Clarification and probing questions (examples)**

- Who exactly was that?
- What was the outcome?
- Who advised you?
- How did you, your child, or your family feel?
- What exactly did you find burdensome or supportive?
- Can you tell me the reasons?

**General follow-up questions guided by the chronology table**

- Do you see any links between the care situation and emotional well-being at particular points in time? If yes, which ones, and why?
- What have you found supportive so far in your child’s care?
- What has been particularly burdensome?
- What ideas, wishes, or suggestions do you have that might have improved your child’s care or supported you as a family?
- Do you feel sufficiently informed, guided, or counselled in dealing with your child’s diagnosis? What do you already know, and what would you like more information about?
- In your experience, is the care pathway clear and self-explanatory, or where do you see general problems, including for other families?
- Do you perceive the care situation as fair?
- How do you currently look to the future?
- Do you have any questions for us?

**Closing**

Thanks and farewell.

**Chronology table for interview preparation**

This table was sent to participants in advance to help them reconstruct the care pathway chronologically, from the first observed concern to the current situation. It served as a memory aid and as an orienting device for the interview. The table was intended solely as an optional private preparation aid; completed copies were not collected or analysed and did not form part of the study data.

| Child’s age / point in time | What happened / was observed (important event or milestone) | How I / we as a family / my child felt at that time (well-being; emojis optional) |
| --- | --- | --- |
|  |  |  |
|  |  |  |
|  |  |  |
|  |  |  |
|  |  |  |
|  |  |  |
|  |  |  |
|  |  |  |

**Development of the supporting interview aid**

During the pilot phase, an emotion diagram was used as an optional reflective element. In the main study, this element was replaced by the chronology table sent to participants in advance in order to better support the reconstruction of relevant stages of the care pathway. The chronology table was intended solely as an optional private preparation aid; completed copies were not collected or analysed and did not form part of the study data. The substantive orientation of the interview guide remained unchanged. Pilot interviews served solely to refine the interview materials and were not included in the present analysis.
