# Supplementary material for: Mothers’ experiences of care pathways for children with speech and developmental concerns in Germany: A qualitative study of navigation, transitions, and communication
Source: PLoS One. 2026 Sep 16;21(9):e0357294. doi: 10.1371/journal.pone.0357294 (PMC13580943; doi:10.1371/journal.pone.0357294)
Supplement: Supplementary file 4 [file pone.0357294.s004.docx]

**S4 File. Comprehensive evidence table.**

**Note.** Interviews were conducted in German. All direct quotations in this supplement were translated into English by the authors. Evidence passages are presented as exemplar material supporting the main analytic claims. Where necessary for clarity and condensation, brief context-based paraphrases or closely condensed renderings are used instead of verbatim quotations. Direct identifiers were removed, and potentially identifying contextual details were generalised or omitted where this did not affect the substantive meaning.

| Overarching analytical unit | Core analytic finding | Variant/contrast | Exemplar evidence passage | Interview |
| --- | --- | --- | --- | --- |
| Early perceptions and triggers of the care pathway | Beginning is framed as early maternal noticing of atypical development | Maternal observation precedes institutional feedback | It became noticeable early on that his language was not developing like that of his siblings. | I10 |
| Early perceptions and triggers of the care pathway | Beginning is marked by an institutional context | Kindergarten functions as the triggering context | That was really when it started in kindergarten. | I8 |
| Early perceptions and triggers of the care pathway | Pressure to act intensifies through the interplay of maternal noticing and institutional feedback | Maternal noticing and kindergarten feedback | The first signs definitely appeared between ages two and three, when we noticed: “Okay, linguistically, the child is not developing as he should” ... and by age three we had already received the first feedback from the kindergarten that the child was not developing in an age-appropriate way, especially in terms of language. | I3 |
| Early perceptions and triggers of the care pathway | Early indications are also articulated in medical contexts | SPZ functions as a medical-institutional prompt | Then we went back to the SPZ ... and they said: “Well, language is somewhat noticeable.” | I6 |
| Early perceptions and triggers of the care pathway | Early concerns initially remain open or deferred | No clear institutional trigger has yet emerged | The U9 check-up is coming up this autumn. Maybe it will be addressed then. But I think less by her [the doctor] and more by me. | I7 |
| Assessment and access as an ambivalent phase of orientation | Early assessment does not necessarily lead to diagnostic clarification | Clarification is deferred with reference to the child being “too young” | At that time they didn’t want to diagnose anything and said it was far too early. | I3 |
| Assessment and access as an ambivalent phase of orientation | Lack of diagnostic clarification is experienced as an ongoing burden | Persistent uncertainty about the problem and its causes | The problem, of course, is that it still hasn’t been diagnosed what kind of language disorder he actually has. So where does it come from? ... the causes have not been clarified. | I10 |
| Assessment and access as an ambivalent phase of orientation | Receiving a diagnosis creates orientation and facilitates next steps | Relief and functional relevance for access | At last it had a name. | I9 |
| Assessment and access as an ambivalent phase of orientation | Diagnoses are experienced as practically effective for the subsequent care pathway | From the mother’s perspective, describing the problem alone is not enough | Over all these years, I have learned that you can achieve much more with, let’s say, solid diagnoses than with merely describing a problem. | I9 |
| Navigation, coordination, and communication along the pathway | Self-coordination emerges in response to multiple parallel demands | Repeated movement across services and institutions becomes burdensome | This diagnostic search, this running from doctor to doctor, from institution to institution. | I9 |
| Navigation, coordination, and communication along the pathway | Lack of navigation support intensifies mothers’ search work | No continuous contact person | You know, there is no single contact person ... you have to find everything out yourself and work everything out for yourself. | I6 |
| Navigation, coordination, and communication along the pathway | Interfaces are experienced as contradictory | Recommendations across services do not translate into coordinated action | CA: early intervention service and SPZ acknowledged the need for both speech and language therapy and occupational therapy, but coordinated implementation did not follow. | I6 |
| Navigation, coordination, and communication along the pathway | Parallel care is structurally restricted | Restriction articulated in the paediatric context | And now it looks like this ... he said: “Either speech therapy or occupational therapy.” I cannot get both at the same time. | I6 |
| Navigation, coordination, and communication along the pathway | Communication can provide orientation and support | Empathic communication and ongoing guidance are experienced as helpful | CA: later service experiences were described as significantly more empathic and as continuing to advise and accompany the family. | I9 |
| Navigation, coordination, and communication along the pathway | Communication is experienced as burdensome when difficulties are reduced to parenting | Attribution and shaming | ... being told that this is all a parenting issue burdens me so much, because it means people do not acknowledge that this child is different, and instead always shift it onto the mother’s parenting. | I3 |
| Navigation, coordination, and communication along the pathway | Mothers wish to be taken seriously rather than reassured with dismissive platitudes | Critique of platitudes and premature everyday explanations | Simply take it seriously. ... Leave out these stock phrases and throw those ready-made beliefs overboard. | I4 |
| Navigation, coordination, and communication along the pathway | Repeated external attributions shift responsibility back to the family | Reduction to a parenting issue | I was told again and again: “Yes, you have to be consistent, you have to go to parenting counselling, he simply needs to be brought up properly.” | I8 |
| Transitions, resources, and future perspectives | Social networks facilitate access and accelerate further steps | Informal contacts function as an access lever | It really makes a world of difference to know someone. | I4 |
| Transitions, resources, and future perspectives | Insurance status influences access to appointments | Faster appointments under private insurance | That is why we are in the fortunate situation that wherever we call, we get an appointment within the next two weeks. | I5 |
| Transitions, resources, and future perspectives | Language and translatability are identified as relevant access conditions | Low-threshold multilingual information | If there were some kind of guide for all these questions, it could probably be translated quite easily into all sorts of languages. | I9 |
| Transitions, resources, and future perspectives | School entry intensifies coordination demands and strain | Repeated interruptions to the school day | At some point, my main task at school was to wait for the school to call and say: “Come and pick up your child.” | I8 |
| Transitions, resources, and future perspectives | Support arrangements at transitions may create additional instability | Pooled support instead of a stable individual arrangement | CA: A pooled school support arrangement was experienced as less stable and less individually responsive than the requested support. | I8 |
| Transitions, resources, and future perspectives | The future trajectory remains partly open and burdensome | Uncertainty about the path ahead | What is still burdensome, I think, is that we don’t know where this journey is going to lead. | I10 |
