# Supplementary material for: Mothers’ experiences of care pathways for children with speech and developmental concerns in Germany: A qualitative study of navigation, transitions, and communication
Source: PLoS One. 2026 Sep 16;21(9):e0357294. doi: 10.1371/journal.pone.0357294 (PMC13580943; doi:10.1371/journal.pone.0357294)
Supplement: Supplementary file 5 [file pone.0357294.s005.docx]

**S5 File. COREQ checklist.**

Consolidated Criteria for Reporting Qualitative Research (32-item checklist)

**Manuscript title:**

Mothers’ experiences of care pathways for children with speech and developmental concerns in Germany: A qualitative study of navigation, transitions, and communication.

| No. | Item | Response for this study | Location in final manuscript / supplement |
| --- | --- | --- | --- |
| 1 | Interviewer/facilitator | Both authors conducted the interviews. | Quality assurance and reflexivity |
| 2 | Credentials | Both interviewers held doctoral degrees. | S5 File (this checklist) |
| 3 | Occupation | At the time of the study, both interviewers were professors and members of the study team. | S5 File (this checklist) |
| 4 | Gender | Both interviewers were female. | S5 File (this checklist) |
| 5 | Experience and training | Both interviewers were therapeutically trained and experienced in professional interviewing and academic research. | Quality assurance and reflexivity |
| 6 | Relationship established | No prior relationship existed between interviewers and participants in the main study interviews included in the present analysis. | S5 File (this checklist) |
| 7 | Participant knowledge of the interviewer | Participants were informed that the interviewers were conducting the study to understand parents’ experiences of educational and health-related care. They were also informed about confidentiality and recording. No additional personal motivations of the interviewers were presented. | ***S3 File – Standardised introduction (script)*** |
| 8 | Interviewer characteristics | Reflexivity was addressed through ongoing reflection on the researchers’ dual role and through team‑internal and external academic discussions of interpretations. | Quality assurance and reflexivity |
| 9 | Methodological orientation and theory | The study used qualitative content analysis informed by Kuckartz and Rädiker, combining deductive and inductive elements. A documented codebook and a case-by-category matrix supported case-based and cross-case analysis. | Data preparation and analysis; Table 2; S1 File; S2 File |
| 10 | Sampling | Sampling was pragmatic yet purposive. The interview component was open to parents and caregivers with parental responsibility; the realised interview sample consisted exclusively of mothers. | Recruitment, sample, and data collection |
| 11 | Method of approach | Recruitment occurred through professional networks, associations, institutional contacts, and social media. Interested survey respondents indicated willingness to be contacted for an interview by providing an email address in the preceding anonymous online survey. | Recruitment, sample, and data collection |
| 12 | Sample size | Eight interviews were included in the present analysis. | Abstract; Study design and reporting; Recruitment, sample, and data collection; Table 1 |
| 13 | Non‑participation | Twenty-two survey respondents provided an email address and thereby indicated willingness to be contacted for an interview. These contact details constituted an opt-in pool rather than a complete sampling frame. Eight interviews were completed; reasons for non-participation or dropout among the remaining contacts were not systematically collected. | Recruitment, sample, and data collection |
| 14 | Setting of data collection | The eight interviews were conducted predominantly by videoconference, with one interview conducted by telephone at the participant’s request. | Recruitment, sample, and data collection |
| 15 | Presence of non‑participants | No non-participants were documented as being present during the interviews. | S5 File (this checklist) |
| 16 | Description of sample | Participant characteristics are reported only in aggregate to protect confidentiality. The realised sample comprised eight mothers. Child age is reported only in broad aggregate form as school-aged children and adolescents, and parent-reported diagnosis or developmental-concern categories are reported in aggregate. Interview duration is reported in the text; no participant-level characteristic profiles are presented. | Study design and reporting; Recruitment, sample, and data collection; Table 1 |
| 17 | Interview guide | A semi-structured interview guide was used; the supporting interview aid was piloted and adapted from an emotion diagram to an optional chronology table. The table served solely as a private preparation and memory aid for participants; completed copies were not collected or analysed and did not form part of the study data. Materials are provided in S3 File. | Recruitment, sample, and data collection; S3 File |
| 18 | Repeat interviews | No repeat interviews were conducted. | Recruitment, sample, and data collection |
| 19 | Audio/visual recording | The interviews were recorded and subsequently transcribed. Seven interviews were conducted via videoconference and one was conducted by telephone. | Recruitment, sample, and data collection; Ethics and data protection |
| 20 | Field notes | No formal field notes were taken during or immediately after the interviews. | S5 File (this checklist) |
| 21 | Duration | Interviews lasted between 19 and 68 minutes, with a median duration of 39 minutes. | Recruitment, sample, and data collection |
| 22 | Data saturation | The analysis proceeded iteratively until central process-related categories recurred across the completed interviews and the coding guide no longer required substantial modification during consensual coding. The study does not claim saturation in a strong quantitative or exhaustive sense. | Data preparation and analysis |
| 23 | Transcripts returned | Transcripts were not returned to participants for comment or correction. | Recruitment, sample, and data collection |
| 24 | Number of data coders | Two researchers coded the material on the basis of the coding guide within a consensus-based interpretive process. Discrepancies in coding, category boundaries, or segmentation were discussed in several rounds of consultation. An exemplary coding comparison was conducted for 50% of one interview and used as a supplementary consistency check. | Quality assurance and reflexivity |
| 25 | Description of the coding tree | A short version of the codebook is presented in Table 2; the full codebook with definitions and anchor examples is provided in S1 File. | Data preparation and analysis; Table 2; S1 File |
| 26 | Derivation of themes | Initial coding was guided by the research questions, the interview guide, and sensitising concepts derived from the health literacy and care pathway literature. Categories and subcategories were iteratively refined in close engagement with the material; four overarching analytical units resulted from later analytic condensation. | Data preparation and analysis; Table 3 |
| 27 | Software | MAXQDA was used to manage the category system, document coding decisions, and support an exemplary coding comparison. | Data preparation and analysis |
| 28 | Participant checking | No systematic participant checking of transcripts or findings was conducted in the main study. | Recruitment, sample, and data collection |
| 29 | Quotations presented | Quotations are identified by interview ID and timestamps or contextual anchors; direct quotations were translated into English. Fuller evidence is provided in S4 File. | Study design and reporting; Results; S4 File |
| 30 | Data and findings consistent | The four analytical units are supported by quotations in the manuscript, the case-by-category matrix in S2 File, and the comprehensive evidence table in S4 File. | Results; S2 File; S4 File |
| 31 | Clarity of major themes | Four overarching analytical units are clearly presented and consistently organised across the Results section, Fig 1, Table 4, and Supporting Information. | Results; Figure 1; Table 4; S2 File; S4 File |
| 32 | Clarity of minor themes | Variation, overlaps, and differing pathway constellations are described in the Results and further documented in S2 File and S4 File. | Results; S2 File; S4 File |

Abbreviations: COREQ = Consolidated Criteria for Reporting Qualitative Research; N/A = not reported in the manuscript / not applicable.
