# Supplementary material for: Mothers’ experiences of care pathways for children with speech and developmental concerns in Germany: A qualitative study of navigation, transitions, and communication
Source: PLoS One. 2026 Sep 16;21(9):e0357294. doi: 10.1371/journal.pone.0357294 (PMC13580943; doi:10.1371/journal.pone.0357294)
Supplement: Supplementary file 6 [file pone.0357294.s006.docx]

Dear author(s)

The manuscript addresses an important and timely topic concerning access to health services and navigation within care systems, and it makes a valuable contribution by foregrounding mothers’ experiences. However, in my view, the manuscript requires limited but necessary revisions to strengthen methodological transparency, define the boundaries of the realised sample more clearly, improve the alignment between the findings and the discussion, and clarify several conceptual and contextual points. I therefore recommend that the manuscript be reconsidered following minor revision.

1. The realised sample consists entirely of families who were receiving occupational therapy and/or speech and language therapy at the time of the survey or within the previous two years (lines 196-203). Because recruitment ran through therapy networks, the study reaches families who had already entered the system, while those who faced the heaviest access barriers and never entered, or dropped out early, are absent by design. The claims about access and navigation (for example lines 722-729 and 748-758) therefore describe families who got in despite barriers rather than those barred from entry. Please name this directly in the limitations (lines 771-780) and qualify the access-related conclusions accordingly.
2. The realised sample consists only of mothers (lines 189, 224), which is noted in the limitations (lines 777-780), yet the title, abstract, and discussion use "parents" throughout. The data carry only the maternal perspective, so statements of the form "parents experience…" reach beyond what the sample shows. Either align the findings consistently with "mothers," or treat the concentration of coordination and gatekeeping work on mothers as a finding in its own right, since it speaks directly to the organisational health literacy framing. At present it is handled only as a limitation, which leaves an analytic point unused.
3. The analysis is described as "qualitative content analysis" (lines 16, 248-264) without specifying the variant. This is an umbrella term, and the procedures differ across the Mayring, Kuckartz, Schreier, and Graneheim & Lundman traditions (inductive category development, deductive category application, summarising versus structuring variants). Please name the specific approach and cite it. Relatedly, please state whether the analysis was inductive, deductive, or combined, and whether health literacy entered as a sensitising concept guiding the coding or only at the interpretive stage. The categories in Table 2 read as largely descriptive, while the link to health literacy is made only in the discussion, so the relationship between the framework and the coding should be made explicit.
4. The interviews were conducted in German and the quotations are presented in English (for example lines 365-370, 405-406, 487-490, and Table 4), but the translation process is not described. Please state who translated the excerpts, whether the translations were checked, and whether the original German wording is available in a supplement. For a study that treats linguistic accessibility as a theme in its own right (lines 491-496), this omission is notable.
5. The intercoder comparison rests on 50% of a single interview, with percentage agreement of about 90% (lines 298-304). The authors qualify this correctly as an indicative measure rather than independent reliability (lines 781-785), which is appropriate, but a figure drawn from half of one of eight interviews is thin when it is positioned as evidence of consistency. There is also some tension between a consensual, interpretive coding logic (lines 259-262) and an agreement/reliability logic, which belong to different traditions. Consider either broadening the agreement check or reframing rigour around consensual coding and the audit trail, with percentage agreement as a secondary indicator.
6. The children range from 6 to 15 years (line 224; I8 and I9 are 15 in Table 1), so some mothers are reconstructing first signs from age 2-3 across more than a decade. Both recall and the fact that the care system itself has changed over that span affect statements of the form "mothers experienced the system as…," since different mothers may be describing the system at different points in time. The retrospective character is acknowledged (lines 231-232), but the length of the recall window and its consequences should be addressed more directly.
7. The discussion is long and aligns the findings with prior literature at many points through "consistent," "similar," and "comparable" (for example lines 559-573, 643-649, 686-692), which supports credibility, but the functional requirements (lines 710-764) are at times only loosely tied to the data and read partly as a restatement of the cited sources. Please make clear which recommendations follow from these eight interviews and which from the wider literature, and where possible link each requirement to a specific finding and, ideally, a quotation. The cautious framing at the end (lines 759-764) is well placed; tightening the section would carry it further.
8. The findings argue that the pathway is non-linear (lines 333-335, 518-520), while the presentation is organised into four units that read as an almost chronological sequence (early perception → assessment → navigation → transitions; lines 271-283, Table 3). This is not a contradiction, but it should be named: state that the four units follow a process or temporal logic rather than a thematic one, while the nodal points can reorder or overlap within cases. This is partly noted in the figure caption (lines 340-344) and should appear in the text as well. Relatedly, the relationship between the seven nodal points (A-G) in Figure 1 and the four analytical units is not immediately clear, and the mapping between them should be made explicit.
9. Several features of the German care system are not introduced sufficiently for non-German readers: the SPZ (lines 380, 451), the U9 check-up (line 383), Frühförderung / early intervention (line 450), the SP3 code (partly explained, lines 222-223), and the "pool" support model (line 502). A short paragraph describing the institutional architecture, including the health-education interface and which body is responsible for what, would help both comprehensibility and transferability.
10. Twenty-two parents left an email address and eight were interviewed (lines 205-208); the reasons the remaining fourteen were not interviewed, and the fact that reasons for non-participation were not collected (line 215), are stated, but the basis for stopping at eight, whether saturation or a pragmatic constraint, should be made explicit. The phrase "the category system had stabilised" (lines 260-262) should be used with care given a sample this small and heterogeneous.
11. The two principal investigators conducted the interviews (line 285) and carried out the coding (line 296), transcripts and findings were not returned to participants (line 236), and the researchers are therapeutically trained (line 286), which carries a possible professional stance toward how the system is portrayed. The reflexivity paragraph (lines 286-292) is somewhat general; it would be more convincing if it addressed how this professional position may have shaped both the interview dynamic and the interpretation.
12. The four research questions (lines 169-180), and Question 4 in particular (support needs and functional requirements), are answered mainly in the discussion rather than in the results. The mapping between the questions and the findings could be made more visible.
