# Supplementary material for: Mothers’ experiences of care pathways for children with speech and developmental concerns in Germany: A qualitative study of navigation, transitions, and communication
Source: PLoS One. 2026 Sep 16;21(9):e0357294. doi: 10.1371/journal.pone.0357294 (PMC13580943; doi:10.1371/journal.pone.0357294)
Supplement: Supplementary file 7 [file pone.0357294.s008.docx]

**Revision table / Response to Reviewers – PONE-D-26-20144**

**Working basis:** original submitted manuscript, final clean revised manuscript (PONE-D-26-20144_Revised_Manuscript_Clean.docx), PLOS ONE decision letter, Reviewer 2 attachment, and final revision package. This table consolidates the editor and reviewer comments and documents how each point was addressed in the revised manuscript and accompanying files.

**Note.** Abbreviations used in the numbering column: J = journal/editorial requirement; R1 = Reviewer 1; R2 = Reviewer 2. Empirical claims are now framed as mothers’ experiences; recruitment and eligibility remain described as open to parents/caregivers with parental responsibility; broader terminology is retained only where it refers to literature, recruitment, eligibility, or the wider care context.

| **No.** | **Source** | **Comment / requirement** | **Manuscript section / document** | **Implementation in revised manuscript / status** | **Response to reviewer/editor** |
| --- | --- | --- | --- | --- | --- |
| J0 | PLOS ONE / Editor | Resubmission package: separate Response to Reviewers, marked-up revised manuscript with tracked changes, and clean manuscript. | Submission package; not a manuscript-content point | Completed for the resubmission package: the clean revised manuscript, the marked-up/track-changes manuscript, and this point-by-point Response to Reviewers have been prepared as separate files for upload. | We submit a point-by-point Response to Reviewers, a clean revised manuscript, and a marked-up version highlighting changes made to the original submission, as requested by the journal. |
| J1 | PLOS ONE / Editor | Ensure that the manuscript meets PLOS ONE style requirements, including file naming. | Full submission package | Completed for the resubmission package: manuscript, figure, checklist, ethics, and Supporting Information file names and upload labels have been harmonised with the revised manuscript. | We prepared the revised manuscript and response materials according to the journal requirements and harmonised the file names and upload labels with the final revised submission package. |
| J2 | PLOS ONE / Editor | Title page must be included within the main manuscript file, not uploaded only as a separate document. | Beginning of revised manuscript | Completed in the final clean manuscript: the manuscript begins with the full title, short title, authors, affiliation, corresponding author, and keywords. | We inserted the title page information at the beginning of the main manuscript file, including title, short title, authors, affiliation, corresponding author, and keywords. |
| J3 | PLOS ONE / Editor; Reviewer 1 | Data availability: full transcripts cannot be publicly shared for ethical/legal reasons; the maximum possible de-identified analytic evidence must be provided. | Data Availability Statement; Methods: Ethics and data protection; Supporting Information | Completed in the final clean manuscript and Supporting Information package: the Data Availability Statement and ethics/data protection section explain the transcript-sharing restriction and identify the de-identified analytic evidence provided as Supporting Information. | We revised the Data Availability Statement and the ethics/data protection section. Full German interview transcripts cannot be made publicly available because detailed narrative accounts may permit indirect identification even after de-identification and because participants did not consent to unrestricted public sharing of complete transcripts. To provide the maximum possible transparency within these constraints, the de-identified minimal analytic dataset underlying the findings is provided as Supporting Information, including the full codebook, the case-by-category matrix, and the comprehensive evidence table. |
| J4 | PLOS ONE / Editor | Ethics statement should appear only in the Methods section and be deleted from any other section. | Methods: Ethics and data protection | Completed in the final clean manuscript: ethics approval, informed consent, data protection, and transcript-sharing restrictions are located in Methods; no separate ethics statement outside Methods was retained. | We ensured that the ethics statement is included in the Methods section only, under “Ethics and data protection”. |
| J5 / R1.2 | PLOS ONE / Editor; Reviewer 1 | Review recommended methodological sources and cite COREQ; Tong et al. (2007) was missing from the reference list. | Methods: Study design and reporting; References; S5 File | Completed in the final clean manuscript: COREQ is cited as [26], Tong et al. was added to the reference list, and the completed checklist is provided as S5 File. | We added the COREQ reference and now cite Tong et al. (2007) in the Methods section. The completed COREQ checklist is provided as S5 File. |
| J6 | PLOS ONE / Editor | Reference list should be complete and correct; retracted articles, if any, must be justified or replaced. | References | Implemented in the revised manuscript: the reference list was checked and updated for completeness and consistency, including the newly cited methodological and reporting references. | We updated the reference list to include the newly cited methodological and reporting references and checked the revised reference list for completeness and consistency with the manuscript citations. |
| R2.2 | Reviewer 2 | The realised sample consists only of mothers, while the original manuscript used “parents” throughout; empirical claims must be aligned with the maternal sample or treated analytically. | Title; Abstract; Introduction; Research Questions; Methods; Results; Discussion; Limitations; Conclusion; Acknowledgments | Completed in the final clean manuscript. The empirical focus is now consistently framed as mothers’ experiences. Recruitment and eligibility are described as open to parents and caregivers with parental responsibility irrespective of gender. Literature and theoretical context passages retain broader terms such as parents, caregivers, or families where this matches the cited sources and avoids an unjustified narrowing to mothers. | We revised the title and wording throughout the manuscript to align empirical claims with the realised interview sample. The final manuscript now states that recruitment was open to parents and caregivers with parental responsibility irrespective of gender, while all interview participants were mothers. We therefore use “mothers” for the realised sample and our empirical findings, while retaining broader terms such as “parents”, “caregivers”, or “families” where they refer to recruitment, eligibility criteria, literature, or the broader care context. The realised maternal sample is also discussed as a limitation and as analytically relevant for interpreting the navigation and coordination work reconstructed in the study. |
| R1.1 / R2.3 | Reviewer 1 and Reviewer 2 | The qualitative content analysis approach should be named and cited; the deductive/inductive logic and the role of health literacy should be clarified. | Abstract; Methods: Data preparation and analysis; References | Completed in the final clean manuscript. The text names Kuckartz and Rädiker, specifies deductive-inductive elements, and frames health literacy as a sensitising perspective rather than a fixed deductive coding framework. | We specified that the analysis followed qualitative content analysis informed by Kuckartz and Rädiker. We clarified that the analysis combined deductive and inductive elements: the research questions and the broader health literacy perspective sensitised the analysis to orientation, comprehensibility, navigation, coordination, communication, and organisational demands, while categories and subcategories were iteratively refined in close engagement with the interview material. We also clarified that health literacy was not applied as a fixed deductive coding framework. |
| R1.3 / R2.4 | Reviewer 1 and Reviewer 2 | The translation process for German interviews and English quotations should be described. | Methods: Recruitment, sample, and data collection; before Data preparation and analysis | Completed in the final clean manuscript. German data collection/transcription, analysis on German transcripts, translation by one author, and checking against the German original by the second author are described. | We added a description of the translation procedure. Interviews were conducted and transcribed in German, and analysis was carried out on the German transcripts. Quotations selected for the manuscript and Supporting Information were translated into English by one author after analysis and checked against the German original by the second author. Translation focused on preserving analytic meaning and readability while protecting participant confidentiality. |
| R1.4 / R2.5 | Reviewer 1 and Reviewer 2 | The intercoder comparison is limited; rigour should be framed around consensual coding and audit trail, with percentage agreement only as a supplementary indicator. | Methods: Quality assurance and reflexivity; Limitations | Completed in the final clean manuscript. The 50% coding comparison is presented as a supplementary consistency check only; no chance-corrected reliability coefficient is claimed. | We revised the quality assurance section to frame the analysis as consensus-based interpretive coding supported by documented coding decisions, case-by-category comparison, linked excerpts, and supplementary materials. The exemplary coding comparison is now presented only as a supplementary consistency check within the project, not as a measure of strictly independent reliability; accordingly, no chance-corrected coefficient was calculated. |
| R1.5 | Reviewer 1 | The consent/permission basis for the preliminary pilot interviews conducted before ethics approval should be clarified. | Methods: Recruitment, sample, and data collection | Completed in the final clean manuscript. The pilot interviews are described as voluntary preliminary interviews for instrument development and procedural refinement; written and oral consent and exclusion from the final analytic dataset are stated. | We clarified that the two preliminary pilot interviews were conducted before ethics approval solely to test and refine the interview aid and practical procedures. Pilot participants were informed about data protection and their rights and provided written and oral consent, including permission to use the pilot conversations for instrument development and scientific purposes. Pilot material was not included in the final analytic dataset, and no pilot quotations, case material, or pilot-derived findings are reported in the manuscript. |
| R1.6 / R2.10 | Reviewer 1 and Reviewer 2 | It should be clarified how 22 interested contacts resulted in eight interviews and why recruitment stopped at eight; strong saturation claims should be avoided. | Methods: Recruitment, sample, and data collection; Methods: Data preparation and analysis | Completed in the final clean manuscript. The 22 contacts are described as survey respondents who provided an email address, i.e. an opt-in pool rather than a complete sampling frame. The manuscript avoids a strong saturation claim and instead describes pragmatic scope, information-rich material, recurring process-related categories, and a stabilised coding guide. | We clarified that the 22 contacts were survey respondents who provided an email address and thereby constituted an opt-in pool for possible follow-up interviews rather than a complete sampling frame from which all eligible families could be systematically followed up. Interviews were organised sequentially within the pragmatic scope of the qualitative interview component. We also clarified that we do not claim saturation in a strong quantitative or exhaustive sense; instead, the completed interviews provided information-rich material for the focused process-oriented analysis, and no further interviews were conducted once central process-related categories recurred across cases and the coding guide no longer required substantial modification during consensual coding. |
| R2.1 / R2.6 / R1.7 / R1.8 | Reviewer 2 and Reviewer 1 | Sample boundaries, access-route limitations, child age range/retrospective recall, diagnostic heterogeneity, and variation in interview duration/depth should be addressed. | Methods: Recruitment/sample; Table 1 note; Limitations | Completed in the final clean manuscript. Limitations state that findings refer to mothers whose families had entered or recently used therapy and do not represent families who never accessed services or disengaged early. Retrospective accounts, broader child age range, changing care structures, diagnostic heterogeneity, and unequal narrative depth are addressed. | We expanded the sample description and limitations. The findings refer to mothers whose families had entered or recently used occupational therapy and/or speech and language therapy services. They do not represent families who never accessed such services, disengaged early, or were not reached through the recruitment pathways. We also added limitations regarding the broader child age range, retrospective accounts and possible changes in care structures over time, diagnostic heterogeneity, and variation in interview duration and narrative depth. |
| R2.7 | Reviewer 2 | Discussion and practical implications should be more clearly linked to the interview data and distinguished from wider literature-supported implications. | Discussion: Practical implications and functional requirements | Completed in the final clean manuscript. Practical implications are explicitly framed as functional requirements derived from mothers’ reconstructions, linked to patterns in the data, and then related to existing literature. The manuscript states that it does not test effectiveness of specific models. | We revised the practical implications section to distinguish more clearly between functional requirements derived from the interview material and broader recommendations supported by the literature. Each implication is now linked to specific patterns in the data before being related to existing research, and the section explicitly states that the study does not test the effectiveness of specific intervention or service models. |
| R2.8 / R2.12 | Reviewer 2 | The relationship between non-linear process logic, the four analytical units, nodal points A–G in Fig. 1, and the research questions should be clearer. | Results: opening paragraph; Fig. 1 caption; Integrative synthesis | Completed in the final clean manuscript. The four analytical units are described as reader-oriented process moments, not as a fixed chronology. Fig. 1 maps A-G to the units, and the integrative synthesis explicitly addresses RQ1-RQ4. | We clarified that the four analytical units provide a reader-oriented structure for presenting recurring process moments and do not imply that all mothers moved through the same stages in the same order. We also added an explicit mapping between the nodal points A–G in Fig. 1 and the four analytical units, and strengthened the integrative synthesis so that it shows how the findings address the four research questions. |
| R2.9 | Reviewer 2 | German care-system terms and institutional interfaces should be briefly explained for international readers. | Introduction; Results where needed; Table 1 note | Completed in the final clean manuscript. The Introduction explains preventive examinations/U9, paediatricians, medical prescriptions for SLT/OT, social paediatric centres, early intervention, daycare/school, and school transitions. Pooled support is explained where it appears. | We added a brief contextual paragraph on the German care context, including preventive child health examinations, paediatricians, prescribed speech and language therapy and occupational therapy, social paediatric centres, early intervention services, and the health–education interface. We also clarified the pooled school support model where it appears in the Results section. |
| R2.11 | Reviewer 2 | Reflexivity should address how the researchers’ professional therapeutic background may have shaped the interview dynamic and interpretation. | Methods: Quality assurance and reflexivity | Completed in the final clean manuscript. The researchers’ therapeutic background is described as both a resource and a possible source of interpretive bias; potential effects on professional terminology, assumed shared background knowledge, and system/therapy-related focus are named. | We expanded the reflexivity section. We now state that both researchers’ therapeutic training and experience were treated as a resource and as a potential source of interpretive bias. We also specify how this background may have shaped the interview interaction and interpretation, for example by encouraging professional terminology, assumed shared background knowledge, or a focus on system- and therapy-related experiences, and we describe how these assumptions were reflected during the research process. |
| R1.Language | Reviewer 1 | The manuscript reads clearly; a few long sentences could be tightened, but this was not considered a barrier to acceptance. | Full manuscript | Completed in the revised manuscript: the manuscript was revised for clarity while prioritising the substantive and methodological issues raised by the reviewers. | We revised the manuscript for clarity while prioritising the substantive and methodological issues raised by the reviewers. |
| J7 | PLOS ONE Editorial Office / administrative check | Remove or sufficiently generalise potentially identifying participant information in the manuscript and Supporting Information. | Abstract; Methods: Recruitment, sample, and data collection; Table 1; Ethics and data protection; Data Availability Statement; S1–S5 | Completed. Participant-level age, interview date, duration, and diagnosis profiles were removed from Table 1 and replaced with aggregate sample information. Child age was generalised to school age and adolescence. Potentially identifying contextual details in the Supporting Information were generalised or omitted. The manuscript and Supporting Information now consistently describe the materials as de-identified and clarify the remaining risk of indirect identification. | We revised the manuscript and all Supporting Information files to further minimise re-identification risk. The participant-level version of Table 1 was replaced with an aggregate description of the sample, and exact age and diagnosis combinations were removed. Potentially identifying contextual details in S1, S2, and S4 were generalised or omitted where this did not affect substantive meaning. We also clarified in the manuscript, S3, and S5 that the chronology table was an optional private preparation aid and that completed copies were neither collected nor analysed. The Data Availability Statement and ethics/data protection section were updated accordingly. |
