# Supplementary material for: Mothers’ experiences of care pathways for children with speech and developmental concerns in Germany: A qualitative study of navigation, transitions, and communication
Source: PLoS One. 2026 Sep 16;21(9):e0357294. doi: 10.1371/journal.pone.0357294 (PMC13580943; doi:10.1371/journal.pone.0357294)
Supplement: Supplementary file 8 [file pone.0357294.s009.docx]

**Manuscript ID:** PONE-D-26-20144R1
**Title:** *Mothers’ experiences of care pathways for children with speech and developmental concerns in Germany: A qualitative study of navigation, transitions, and communication*

Dear Academic Editor and Reviewers,

We sincerely thank the Academic Editor and both reviewers for their careful assessment of our revised manuscript and for their positive evaluation of the revisions made in the previous round. We are particularly grateful for the two remaining suggestions from Reviewer 2, which helped us further clarify the manuscript. We have addressed both points as described below.

**Reviewer 2 – Comment 1**

**Comment:**
The maternal composition of the sample is described as analytically relevant because the coordination work was reconstructed from a maternal position. This is stated but not developed. Please either add a short sentence linking it to existing work on how care and coordination work is distributed within families, or adjust the wording so that the claim matches what the analysis actually does.

**Response:**
Thank you for this important clarification. We agreed that the previous wording could be interpreted as making a broader claim about gendered caregiving roles than is supported by our data. We therefore chose the second option suggested by the reviewer and revised the passage so that it more closely reflects the scope of the analysis.

The revised text in the **Limitations** section now reads:

“Accordingly, the navigation and coordination work reconstructed in this study should be interpreted specifically as described from mothers’ perspectives rather than as representative of parental caregiving more broadly.”

We retained the subsequent clarification that the study was not designed to compare gendered caregiver roles and that such comparisons remain beyond its scope. This revision preserves the empirical observation that the realised interview sample consisted exclusively of mothers while avoiding conclusions about the broader distribution of caregiving or coordination work within families.

**Reviewer 2 – Comment 2**

**Comment:**
The reason for not conducting further interviews is given twice in almost the same form. One of the two can be removed.

**Response:**
Thank you for pointing out this redundancy. We removed the earlier sentence stating that the analysis proceeded until central themes recurred and the category system no longer required substantial modification.

We retained the subsequent passage in the **Methods – Data preparation and analysis** section because it directly explains the decision not to conduct further interviews while also preserving our deliberately cautious wording regarding saturation:

“We do not claim saturation in a strong quantitative or exhaustive sense. Rather, within the pragmatic scope of the qualitative interview component, the completed interviews provided information-rich material for the focused process-oriented analysis, and no further interviews were conducted once the central process-related categories recurred across cases and the coding guide no longer required substantial modification during consensual coding.”

This removes the repetition without changing the methodological interpretation of the sampling process.

We thank the Academic Editor and the reviewers again for their constructive comments and for the opportunity to further refine the manuscript.

Sincerely,

Stephanie Rupp
on behalf of all authors
