# Supplementary material for: Mothers’ experiences of care pathways for children with speech and developmental concerns in Germany: A qualitative study of navigation, transitions, and communication
Source: PLoS One. 2026 Sep 16;21(9):e0357294. doi: 10.1371/journal.pone.0357294 (PMC13580943; doi:10.1371/journal.pone.0357294)
Supplement: Peer review history

# Peer review: Decision Letter 0

**DOI:** 10.1371/journal.pone.0357294.r001

**Authors:** Gökhan Töret

28 Jun 2026

Parents’ experiences of care pathways for children with speech and developmental concerns in Germany: A qualitative study of navigation, transitions, and communication

PLOS One

Dear Dr. Rupp,

Thank you for submitting your manuscript to PLOS ONE. After careful consideration, we feel that it has merit but does not fully meet PLOS ONE’s publication criteria as it currently stands. Therefore, we invite you to submit a revised version of the manuscript that addresses the points raised during the review process.

Please submit your revised manuscript by Aug 12 2026 11:59PM. If you will need more time than this to complete your revisions, please reply to this message or contact the journal office at plosone@plos.org. When you're ready to submit your revision, log on to https://www.editorialmanager.com/pone/ and select the 'Submissions Needing Revision' folder to locate your manuscript file.

Please include the following items when submitting your revised manuscript:

- A letter that responds to each point raised by the academic editor and reviewer(s). You should upload this letter as a separate file labeled 'Response to Reviewers'.
- A marked-up copy of your manuscript that highlights changes made to the original version. You should upload this as a separate file labeled 'Revised Manuscript with Track Changes'.
- An unmarked version of your revised paper without tracked changes. You should upload this as a separate file labeled 'Manuscript'.

If you would like to make changes to your financial disclosure, please include your updated statement in your cover letter. Guidelines for resubmitting your figure files are available below the reviewer comments at the end of this letter.

If applicable, we recommend that you deposit your laboratory protocols in protocols.io to enhance the reproducibility of your results. Protocols.io assigns your protocol its own identifier (DOI) so that it can be cited independently in the future. For instructions see: https://journals.plos.org/plosone/s/submission-guidelines#loc-laboratory-protocols. Additionally, PLOS ONE offers an option for publishing peer-reviewed Lab Protocol articles, which describe protocols hosted on protocols.io. Read more information on sharing protocols at https://plos.org/protocols?utm_medium=editorial-email&utm_source=authorletters&utm_campaign=protocols.

As the corresponding author, your ORCID iD is verified in the submission system and will appear in the published article. PLOS supports the use of ORCID, and we encourage all coauthors to register for an ORCID iD and use it as well. Please encourage your coauthors to verify their ORCID iD within the submission system before final acceptance, as unverified ORCID iDs will not appear in the published article. *Only*  the individual author can complete the verification step; PLOS staff *cannot*  verify ORCID iDs on behalf of authors.

We look forward to receiving your revised manuscript.

Kind regards,

Gökhan Töret

Academic Editor

PLOS One

Journal Requirements:

When submitting your revision, we need you to address these additional requirements.

1.Please ensure that your manuscript meets PLOS ONE's style requirements, including those for file naming. The PLOS ONE style templates can be found at

https://journals.plos.org/plosone/s/file?id=wjVg/PLOSOne_formatting_sample_main_body.pdf and

https://journals.plos.org/plosone/s/file?id=ba62/PLOSOne_formatting_sample_title_authors_affiliations.pdf

2. Please ensure that you include a title page within your main document. We do appreciate that you have a title page document uploaded as a separate file, however, as per our author guidelines (http://journals.plos.org/plosone/s/submission-guidelines#loc-title-page) we do require this to be part of the manuscript file itself and not uploaded separately.

Could you therefore please include the title page into the beginning of your manuscript file itself, listing all authors and affiliations.

3. In the online submission form, you indicated that “The minimal data set required to assess the analytic basis of the findings is

publicly available within the manuscript and its Supporting Information files.

This includes the study codebook (S1) and de identified evidence tables and

matrices (S2–S4) containing the interview excerpts underlying the main analytic

claims.

Full interview transcripts are not publicly available because, even after

de identification, qualitative narrative data of this kind may contain potentially

identifying information about participants, children, family contexts, and care

trajectories. Public sharing of full transcripts would therefore risk

compromising participant confidentiality and would not be consistent with the

conditions of ethical approval and informed consent.

De identified additional study materials may be made available to qualified

researchers for scientifically sound reuse purposes upon reasonable request.

Such requests should be directed to the Research Office of IU International

University of Applied Sciences (forschung@iu.org), which acts as the formal data

access point. Access decisions will be made in accordance with ethical approval,

informed consent, and applicable data protection regulations.

This controlled access approach ensures an appropriate balance between data

transparency and the protection of participant confidentiality.”

All PLOS journals now require all data underlying the findings described in their manuscript to be freely available to other researchers, either 1. In a public repository, 2. Within the manuscript itself, or 3. Uploaded as supplementary information.

This policy applies to all data except where public deposition would breach compliance with the protocol approved by your research ethics board. If your data cannot be made publicly available for ethical or legal reasons (e.g., public availability would compromise patient privacy), please explain your reasons on resubmission and your exemption request will be escalated for approval.

4. Your ethics statement should only appear in the Methods section of your manuscript. If your ethics statement is written in any section besides the Methods, please move it to the Methods section and delete it from any other section. Please ensure that your ethics statement is included in your manuscript, as the ethics statement entered into the online submission form will not be published alongside your manuscript.

5. If the reviewer comments include a recommendation to cite specific previously published works, please review and evaluate these publications to determine whether they are relevant and should be cited. There is no requirement to cite these works unless the editor has indicated otherwise.

6. Please review your reference list to ensure that it is complete and correct. If you have cited papers that have been retracted, please include the rationale for doing so in the manuscript text, or remove these references and replace them with relevant current references. Any changes to the reference list should be mentioned in the rebuttal letter that accompanies your revised manuscript. If you need to cite a retracted article, indicate the article’s retracted status in the References list and also include a citation and full reference for the retraction notice.

[Note: HTML markup is below. Please do not edit.]

Reviewer's Responses to Questions

**Comments to the Author**

1. Is the manuscript technically sound, and do the data support the conclusions?

Reviewer #1: Partly

Reviewer #2: Partly

**********

2. Has the statistical analysis been performed appropriately and rigorously?

Reviewer #1: N/A

Reviewer #2: Yes

**********

3. Have the authors made all data underlying the findings in their manuscript fully available?

The PLOS Data policy

Reviewer #1: Yes

Reviewer #2: Yes

**********

4. Is the manuscript presented in an intelligible fashion and written in standard English?

Reviewer #1: Yes

Reviewer #2: Yes

**********

Reviewer #1: This qualitative interview study reconstructs how mothers of children with speech, communication, and developmental concerns in Germany experience the care and support pathway, from the first signs of concern through the uptake or coordination of services. It is the interview component of the EGUBE project. The claims are deliberately modest: the aim is not to measure frequencies or care quality, but to reconstruct process-related demands and the conditions parents associate with burden and relief. Read against that aim, the study is methodologically sound in most respects and its conclusions are supported by the quoted material. A few reporting gaps should be closed before publication. My recommendation is minor revision.

Strengths. The four research questions are stated clearly (lines 169-180). A COREQ checklist is provided (S5). The codebook (S1), case-by-category matrix (S2), and evidence tables (S4) are documented, and the findings can be traced back to this material. The dual role of the researchers as therapists and study leads is addressed in a dedicated reflexivity section (lines 284-307). Ethics approval, trial registration (DRKS00034313), and written informed consent are reported (lines 308-327). The limitations are stated honestly (lines 765-787). Withholding full transcripts on confidentiality grounds, with access routed through a non-author institutional point (the Research Office), is consistent with the exception permitted under the PLOS data policy.

Points to address.

The qualitative content analysis approach should be named and cited. The method is labelled only as "qualitative content analysis" (line 16; lines 248-264) and described through MAXQDA, category development, and consensual coding, but the manuscript does not state which tradition it draws on (for instance Mayring, Kuckartz, or Schreier) and gives no methodological reference. The analysis should be tied to a named framework with the appropriate citation.

COREQ should be cited. The checklist is mentioned (lines 190-191), but Tong et al. (2007) does not appear in the reference list and should be added.

The translation of the quotations should be described. The interviews were conducted in German, yet all direct quotations in Table 4 and the Results are in English (for example lines 365-384). The Methods describe only transcription (lines 245-247) and say nothing about who translated the excerpts or how (for example back-translation or a check by a second reader). Since the quotations carry the evidential weight in this study, a short account of the translation procedure is needed.

Intercoder agreement is limited and should be presented as such. Only 50% of one interview was double-coded, and percentage agreement (about 90%) was calculated, with no chance-corrected coefficient such as kappa (lines 298-304). The authors already describe this honestly as an indicative measure rather than strict independent reliability, which is appropriate. Reporting a chance-corrected value on the segment that was already double-coded, or strengthening the rationale for this choice, would make the section more convincing.

The consent basis for the pilot interviews should be clarified. Two pilot interviews were conducted before ethics approval (line 209; approval at lines 309-310). Pilot data were excluded and the pilots are framed as material development, so the reported study is post-approval and compliant. A single sentence on the permission or consent basis for these pre-approval interviews would settle a question an ethics editor may otherwise raise.

The selection of the eight interviews should be specified. Twenty-two people consented to be contacted (lines 205-207) and eight were interviewed (lines 207-208). The phrase "pragmatic yet purposive" (line 199) does not convey the selection criteria; a brief statement of how these eight were chosen would help. The absence of systematically collected reasons for non-participation is already noted (lines 213-215).

Minor points. The diagnostic profile in Table 1 is heterogeneous (developmental language disorder alongside hyperkinetic disorder, ADHD, Asperger syndrome, and pervasive developmental disorder). The "developmental concerns" framing in the title accommodates this, but a sentence on its implications for the analysis would be useful. Some interviews are short (I7 about 19 minutes); this is reported transparently and is acceptable, though a brief note on data depth could be added. Figure 1 is presented as a heuristic, and the text rightly states that it is not a fixed sequence (lines 340-344), which is fine as it stands.

Language. The manuscript reads clearly and is intelligible throughout. A few long sentences could be tightened, but this is not a barrier to acceptance.

Reviewer #2: Dear author(s)

I recommend minor revision, as the manuscript addresses an important topic but requires clarification of the sample boundaries, analytic approach, translation procedures, and the alignment between findings, discussion, and conclusions.

**********

what does this mean?). If published, this will include your full peer review and any attached files.

If you choose “no”, your identity will remain anonymous but your review may still be made public.

**Do you want your identity to be public for this peer review?**  For information about this choice, including consent withdrawal, please see our Privacy Policy

Reviewer #1: **Yes:**  Ali KAYA

Reviewer #2: No

**********

[NOTE: If reviewer comments were submitted as an attachment file, they will be attached to this email and accessible via the submission site. Please log into your account, locate the manuscript record, and check for the action link "View Attachments". If this link does not appear, there are no attachment files.]

To ensure your figures meet our technical requirements, please review our figure guidelines: https://journals.plos.org/plosone/s/figures

You may also use PLOS’s free figure tool, NAAS, to help you prepare publication quality figures: https://journals.plos.org/plosone/s/figures#loc-tools-for-figure-preparation.

NAAS will assess whether your figures meet our technical requirements by comparing each figure against our figure specifications.

Attachment Submitted filename: Min- Rev.-PONE-D-26-20144.docx

# Author comment: Author response to Decision Letter 1

**DOI:** 10.1371/journal.pone.0357294.r002

23 Jul 2026

Please see the separately uploaded file “PONE-D-26-20144_Response_to_Reviewers_Table.docx” for our detailed point-by-point response to all comments raised by the Academic Editor and the reviewers.

Attachment Submitted filename: PONE-D-26-20144_Response_to_Reviewers_Table_neu.docx

# Peer review: Decision Letter 1

**DOI:** 10.1371/journal.pone.0357294.r003

**Authors:** Gökhan Töret

6 Aug 2026

Dear Dr. Rupp,

In the Discussion, the manuscript states that the exclusively maternal composition of the sample is analytically relevant because the coordination work was reconstructed from a maternal position. Please either add a brief sentence linking this point to existing literature on the distribution of care and coordination work within families or revise the wording so that the claim more closely reflects what the analysis demonstrates.

In the Methods section, the rationale for not conducting additional interviews is presented twice in very similar terms. Please remove one of these passages to avoid unnecessary repetition.

Once these minor revisions have been addressed, I think being able to proceed with the editorial decision without further external review.

Please submit your revised manuscript by Sep 20 2026 11:59PM. If you will need more time than this to complete your revisions, please reply to this message or contact the journal office at plosone@plos.org. When you're ready to submit your revision, log on to https://www.editorialmanager.com/pone/ and select the 'Submissions Needing Revision' folder to locate your manuscript file.

- A letter that responds to each point raised by the academic editor and reviewer(s). You should upload this letter as a separate file labeled 'Response to Reviewers'.
- A marked-up copy of your manuscript that highlights changes made to the original version. You should upload this as a separate file labeled 'Revised Manuscript with Track Changes'.
- An unmarked version of your revised paper without tracked changes. You should upload this as a separate file labeled 'Manuscript'.

If you would like to make changes to your financial disclosure, please include your updated statement in your cover letter. Guidelines for resubmitting your figure files are available below the reviewer comments at the end of this letter.

If applicable, we recommend that you deposit your laboratory protocols in protocols.io to enhance the reproducibility of your results. Protocols.io assigns your protocol its own identifier (DOI) so that it can be cited independently in the future. For instructions see: https://journals.plos.org/plosone/s/submission-guidelines#loc-laboratory-protocols. Additionally, PLOS ONE offers an option for publishing peer-reviewed Lab Protocol articles, which describe protocols hosted on protocols.io. Read more information on sharing protocols at https://plos.org/protocols?utm_medium=editorial-email&utm_source=authorletters&utm_campaign=protocols.

As the corresponding author, your ORCID iD is verified in the submission system and will appear in the published article. PLOS supports the use of ORCID, and we encourage all coauthors to register for an ORCID iD and use it as well. Please encourage your coauthors to verify their ORCID iD within the submission system before final acceptance, as unverified ORCID iDs will not appear in the published article. *Only*  the individual author can complete the verification step; PLOS staff *cannot*  verify ORCID iDs on behalf of authors.

We look forward to receiving your revised manuscript.

Kind regards,

Gökhan Töret

Academic Editor

PLOS One

Journal Requirements:

1. If the reviewer comments include a recommendation to cite specific previously published works, please review and evaluate these publications to determine whether they are relevant and should be cited. There is no requirement to cite these works unless the editor has indicated otherwise.

2. Please review your reference list to ensure that it is complete and correct. If you have cited papers that have been retracted, please include the rationale for doing so in the manuscript text, or remove these references and replace them with relevant current references. Any changes to the reference list should be mentioned in the rebuttal letter that accompanies your revised manuscript. If you need to cite a retracted article, indicate the article’s retracted status in the References list and also include a citation and full reference for the retraction notice.

[Note: HTML markup is below. Please do not edit.]

Reviewers' comments:

Reviewer's Responses to Questions

**Comments to the Author**

Reviewer #1: All comments have been addressed

Reviewer #2: All comments have been addressed

**********

2. Is the manuscript technically sound, and do the data support the conclusions?

Reviewer #1: Yes

Reviewer #2: Yes

**********

3. Has the statistical analysis been performed appropriately and rigorously?

Reviewer #1: N/A

Reviewer #2: Yes

**********

4. Have the authors made all data underlying the findings in their manuscript fully available?

The PLOS Data policy

Reviewer #1: Yes

Reviewer #2: Yes

**********

5. Is the manuscript presented in an intelligible fashion and written in standard English?

Reviewer #1: Yes

Reviewer #2: Yes

**********

Reviewer #1: Following the implementation of the requisite revisions, the manuscript is now suitable for publication. I express my sincere appreciation for this highly informative contribution regarding Germany.

Reviewer #2: The empirical framing is now limited to mothers throughout the title, abstract, and main text, while broader terms are kept only for recruitment, eligibility, and cited literature (for example, lines 240-246 and 725-730). The analytic approach is named as qualitative content analysis informed by Kuckartz and Rädiker, and the text now states that the analysis combined deductive and inductive elements and that health literacy served as a sensitising perspective rather than a fixed coding framework (lines 317-324). The translation of the German interviews and the English quotations is described (lines 308-315). The intercoder comparison is now presented as a supplementary consistency check, and no chance-corrected coefficient is claimed (lines 394-401). The access-route limitation is stated directly, including the point that barriers may be at least as relevant for families the study did not reach (lines 886-896). The practical implications are each grounded in the interview material before being related to the literature (lines 823-879). The German care context, the non-linear framing, the mapping of nodal points A -G to the four analytical units, and the mapping to the four research questions are all addressed (lines 70–83; 432-436; 445-449; 628-639).

two minor points remain. None of them requires a further round of review.

1. The maternal composition of the sample is described as analytically relevant because the coordination work was reconstructed from a maternal position (lines 918-921). This is stated but not developed. Please either add a short sentence linking it to existing work on how care and coordination work is distributed within families, or adjust the wording so that the claim matches what the analysis actually does.

2. The reason for not conducting further interviews is given twice in almost the same form (lines 341-343 and 346-348). One of the two can be removed.

**********

what does this mean?). If published, this will include your full peer review and any attached files.

If you choose “no”, your identity will remain anonymous but your review may still be made public.

**Do you want your identity to be public for this peer review?**  For information about this choice, including consent withdrawal, please see our Privacy Policy

Reviewer #1: **Yes:**  Ali KAYA

Reviewer #2: No

**********

[NOTE: If reviewer comments were submitted as an attachment file, they will be attached to this email and accessible via the submission site. Please log into your account, locate the manuscript record, and check for the action link "View Attachments". If this link does not appear, there are no attachment files.]

To ensure your figures meet our technical requirements, please review our figure guidelines: https://journals.plos.org/plosone/s/figures

You may also use PLOS’s free figure tool, NAAS, to help you prepare publication quality figures: https://journals.plos.org/plosone/s/figures#loc-tools-for-figure-preparation.

NAAS will assess whether your figures meet our technical requirements by comparing each figure against our figure specifications.

# Author comment: Author response to Decision Letter 2

**DOI:** 10.1371/journal.pone.0357294.r004

12 Aug 2026

Thank you for your careful review and for the two remaining suggestions. We have addressed both points in the revised manuscript.

First, we revised the wording in the Limitations section to clarify that the navigation and coordination work reconstructed in this study reflects mothers’ perspectives and should not be interpreted as representative of parental caregiving more broadly. We retained the clarification that the study was not designed to compare gendered caregiver roles.

Second, we removed the redundant sentence in the Methods section concerning the recurrence of central themes and stability of the coding guide, while retaining the subsequent, more precise explanation for why no further interviews were conducted.

A detailed point-by-point response is provided in the separate “Response to Reviewers” file.

Attachment Submitted filename: PONE-D-26-20144_Revised_2_Response to Reviewers.docx

# Editor report: Decision Letter 2

**DOI:** 10.1371/journal.pone.0357294.r005

**Authors:** Gökhan Töret

17 Aug 2026

Mothers’ experiences of care pathways for children with speech and developmental concerns in Germany: A qualitative study of navigation, transitions, and communication

PONE-D-26-20144R2

Dear Dr. Rupp,

We’re pleased to inform you that your manuscript has been judged scientifically suitable for publication and will be formally accepted for publication once it meets all outstanding technical requirements.

Within one week, you’ll receive an e-mail detailing the required amendments. When these have been addressed, you’ll receive a formal acceptance letter and your manuscript will be scheduled for publication.

An invoice will be generated when your article is formally accepted. Please note, if your institution has a publishing partnership with PLOS and your article meets the relevant criteria, all or part of your publication costs will be covered. Please make sure your user information is up-to-date by logging into Editorial Manager at Editorial Manager® and clicking the ‘Update My Information' link at the top of the page. For questions related to billing, please contact billing support.

If your institution or institutions have a press office, please notify them about your upcoming paper to help maximize its impact. If they’ll be preparing press materials, please inform our press team as soon as possible -- no later than 48 hours after receiving the formal acceptance. Your manuscript will remain under strict press embargo until 2 pm Eastern Time on the date of publication. For more information, please contact onepress@plos.org.

Kind regards,

Gökhan Töret

Academic Editor

PLOS One

Additional Editor Comments (optional):

Reviewers' comments:

# Editor report: Acceptance letter

**DOI:** 10.1371/journal.pone.0357294.r006

**Authors:** Gökhan Töret

PONE-D-26-20144R2

PLOS One

Dear Dr. Rupp,

I'm pleased to inform you that your manuscript has been deemed suitable for publication in PLOS One. Congratulations! Your manuscript is now being handed over to our production team.

At this stage, our production department will prepare your paper for publication. This includes ensuring the following:

* All references, tables, and figures are properly cited

* All relevant supporting information is included in the manuscript submission,

* There are no issues that prevent the paper from being properly typeset

You will receive further instructions from the production team, including instructions on how to review your proof when it is ready. Please keep in mind that we are working through a large volume of accepted articles, so please give us a few days to review your paper and let you know the next and final steps.

Lastly, if your institution or institutions have a press office, please let them know about your upcoming paper now to help maximize its impact. If they'll be preparing press materials, please inform our press team within the next 48 hours. Your manuscript will remain under strict press embargo until 2 pm Eastern Time on the date of publication. For more information, please contact onepress@plos.org.

You will receive an invoice from PLOS for your publication fee after your manuscript has reached the completed accept phase. If you receive an email requesting payment before acceptance or for any other service, this may be a phishing scheme. Learn how to identify phishing emails and protect your accounts at https://explore.plos.org/phishing.

If we can help with anything else, please email us at customercare@plos.org.

Thank you for submitting your work to PLOS One and supporting open access.

Kind regards,

PLOS ONE Editorial Office Staff

on behalf of

Dr. Gökhan Töret

Academic Editor

PLOS One
